# Hsc70/Stub1 promotes the removal of individual oxidatively stressed peroxisomes

**DOI:** 10.1038/s41467-020-18942-3

**Published:** 2020-10-19

**Authors:** Bo-Hua Chen, Yao-Jen Chang, Steven Lin, Wei Yuan Yang

**Affiliations:** 1 Chemical Biology and Molecular Biophysics Program, Taiwan International Graduate Program, Academia Sinica, Taipei, 115 Taiwan; 2 Institute of Biological Chemistry, Academia Sinica, Taipei, 115 Taiwan; 3 Institute of Bioinformatics and Structural Biology, National Tsing Hua University, Hsinchu, Taiwan; 4 Institute of Biochemical Sciences, College of Life Sciences, National Taiwan University, Taipei, 106 Taiwan

**Keywords:** Pexophagy, Peroxisomes, Ubiquitylation

## Abstract

Peroxisomes perform beta-oxidation of branched and very-long chain fatty acids, which leads to the formation of reactive oxygen species (ROS) within the peroxisomal lumen. Peroxisomes are therefore prone to ROS-mediated damages. Here, using light to specifically and acutely induce ROS formation within the peroxisomal lumen, we find that cells individually remove ROS-stressed peroxisomes through ubiquitin-dependent pexophagy. Heat shock protein 70 s mediates the translocation of the ubiquitin E3 ligase Stub1 (STIP1 Homology and U-Box Containing Protein 1) onto oxidatively-stressed peroxisomes to promote their selective ubiquitination and autophagic degradation. Artificially targeting Stub1 to healthy peroxisomes is sufficient to trigger pexophagy, suggesting a key role Stub1 plays in regulating peroxisome quality. We further determine that Stub1 mutants found in Ataxia patients are defective in pexophagy induction. Dysfunctional peroxisomal quality control may therefore contribute to the development of Ataxia.

## Abstract (web summary)

Pexophagy removes damaged peroxisomes, which are particularly prone to ROS formation. Here, the authors use light to induce peroxisome turnover by local ROS generation, showing STUB1 translocation is critical and might contribute to human disease.

## Introduction

Peroxisomes carry out a plethora of functions within a cell, including the oxidation of fatty acids, as well as the synthesis of a variety of lipids. Peroxisomal functions are essential: individuals lacking adequate number of peroxisomes, either due to defective peroxisome biogenesis, or uncontrolled peroxisome turnover, suffers from diseases broadly defined as the peroxisome biogenesis disorders^1–5^. Dysfunctional peroxisomal proteins can also result in a series of symptoms termed the peroxisomal enzyme deficiencies^6^. Metabolism within the peroxisomal lumen are known to generate a variety of reactive oxygen species (ROS), thereby making peroxisomes susceptible to ROS-induced damages^7,8^. To counter these ROS-induced stresses, there needs to be adequate peroxisomal quality control programs in place for the cells to avoid disorders associated with peroxisomal dysfunction.

One pathway that may be able to perform the quality control at the level of individual peroxisomes is pexophagy, the selective turnover of peroxisomes through autophagy^9^. Indeed, multiple cellular organelles have been reported to be quality controlled through autophagy^10^. On the other hand, during changes in metabolism and environmental conditions, cells are also known to alter the number of cellular peroxisomes through pexophagy^3,11–13^. Furthermore, studies that artificially conjugated ubiquitin to peroxisomal proteins also induced pexophagy^14^. Understanding if and how pexophagy can specifically turnover ROS-stressed peroxisomes will allow one to eventually unveil cellular phenotypes associated with the defective peroxisomal quality control.

Here, we report a form of ubiquitin-dependent pexophagy that selectively clears away ROS-stressed peroxisomes. Heat shock protein 70 s (Hsp70 and Hsc70) allows the ubiquitin E3 ligase Stub1 (STIP1 homology and U-box containing protein 1) to recognize and ubiquitinate ROS-stressed peroxisomes, leading to their turnover by autophagy. Remarkably, optogenetically targeting Stub1 alone onto functional peroxisomes is sufficient to trigger peroxisome ubiquitination, as well as their degradation through autophagy. This suggests a key role Stub1 plays in signaling pexophagy. Moreover, we found that Stub1 mutants discovered in Ataxia patients are deficient in their ability to trigger this form of pexophagy, suggesting an interesting link between defective peroxisomal quality control and Ataxia.

## Results

### Stressing peroxisomes with ROS through peroxisome-targeted KillerRed

**Fig. 1:**
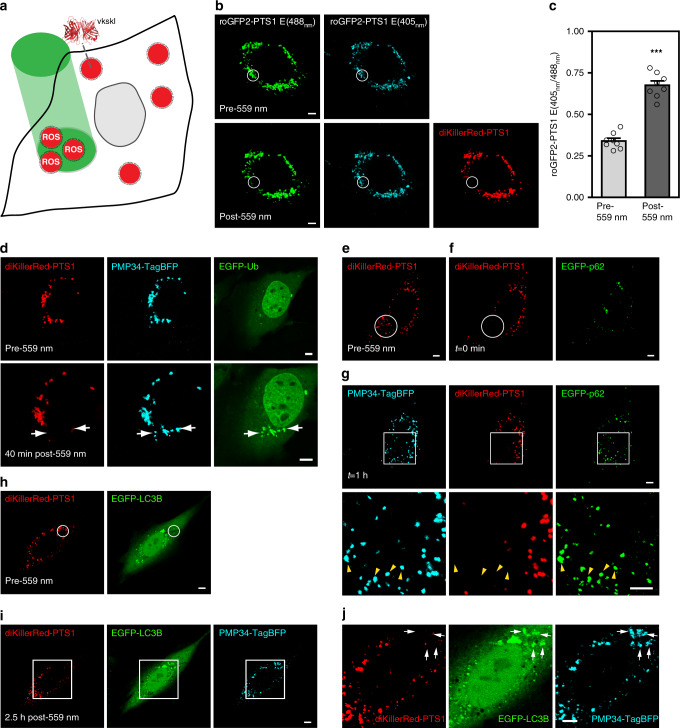
Light-assisted peroxisomal ROS production. **a** Schematic of how we trigger peroxisomal ROS generation by 559 nm light. **b**, **c** The peroxisome redox sensor roGFP2-PTS1 was used to confirm peroxisomal ROS production following 559 nm illumination. **b** Peroxisomes within the white circular region of a NIH3T3 cell expressing diKillerRed-PTS1 and roGFP2-PTS1 were illuminated with 559 nm light (top panels: before 559 nm light illumination; bottom panels: after 559 nm light illumination). Left: roGFP2-PTS1 emission with 488 nm excitation (green), middle: roGFP2-PTS1 emission with 405 nm excitation (cyan). Right: immediate loss of diKillerRed-PTS1 emission following 559 nm illumination. **c** Quantified changes in roGFP2-PTS1 emission ratios (405/488 nm) following 559 nm illumination (in NIH3T3 cells expressing diKillerRed-PTS1 and roGFP2-PTS1). A 559 nm illumination increased the mean 405/488 nm emission ratio for roGFP2-PTS1 (*n* = 8 cells). Error bars displayed on graphs represent the mean + SEM. ***P = 1.72E−05 (one-tailed paired samples *t* test). **d** Peroxisomes indicated by the white arrows in a NIH3T3 cell expressing EGFP-Ub, PMP34-TagBFP, and diKillerRed-PTS1 were illuminated with 559 nm light (top panels), leading to the immediate loss of their diKillerRed-PTS1 fluorescence. EGFP-Ub later specifically accumulated on 559 nm illuminated peroxisomes (bottom panels: 40 min after 559 nm illumination, *n* = 31 cells). **e**–**g** A NIH3T3 cell expressing diKillerRed-PTS1, EGFP-p62, and PMP34-TagBFP was illuminated with 559 nm at the white circular region, leading to the immediate loss in diKillerRed-PTS1 fluorescence. **g** EGFP-p62 later accumulated onto the ROS-stressed peroxisomes (bottom panels: magnified view of the white square region in the top panels. Yellow arrowheads indicate EGFP-p62 on damaged peroxisomes; *n* = 13 cells). **h**, **i** A NIH3T3 cell expressing diKillerRed-PTS1, EGFP-LC3B, and PMP34-TagBFP was illuminated with 559 nm at the white circular region, leading to the local appearance of EGFP-LC3B puncta in **i**. **j** Magnified view of the white square region in **i**. EGFP-LC3B specifically accumulated on 559 nm illuminated peroxisomes (white arrows, *n* = 33 cells). All scale bars, 5 µm. Source data are provided as a Source data file.

We utilized organelle-targeting photosensitizers in conjunction with light illumination to spatially elicit ROS generation to probe how cells handle ROS-stressed peroxisomes. This framework has previously allowed the probing of multiple organelle quality control pathways^15–18^. Here, our design involved the use of a KillerRed tandem dimer diKillerRed (KillerRed has a tendency to self-dimerize^19^, so a tandem dimer was made to improve its physical properties) targeted into the peroxisomal lumen (appended with VKSKL to the C-terminus, a peroxisome targeting sequence (PTS1)), as illustrated in Fig. 1a (ref. ^20^). We validated that diKillerRed-PTS1 properly localized into peroxisomes in NIH3T3 cells, the model cell line used in this study (Fig. 1d, top panels). We then utilized roGFP2-PTS1, a ratiometric fluorescent sensor that reports the redox state within the peroxisomal lumen, to probe the consequence of diKillerRed-PTS1 excitation (using 559 nm illumination)^21^. When oxidized, roGFP2’s excitation maximum shifts from 490 to 400 nm. Increase in the ratios between 405 and 488 nm excited roGFP2-PTS1 emissions therefore reports augmented oxidative stress within peroxisomes. Our roGFP2-PTS1 results (Fig. 1b, c) confirmed that our scheme can allow one to specifically ROS stress a subset of cellular peroxisomes.

### Ubiquitin-dependent pexophagy on ROS-stressed peroxisomes

Using our scheme, and with the help of EGFP-tagged reporter constructs, we found that ROS-stressed peroxisomes (illuminated with 559 nm light) accumulated ubiquitination (together with a loss of diKillerRed-PTS1 fluorescence due to photobleaching), a characteristic response frequently observed in organelle quality control pathways (Fig. 1d, bottom panels, white arrows)^10^. Un-illuminated peroxisomes were not affected. In addition to ubiquitination, ROS-stressed peroxisomes also accumulated the autophagy adaptor protein p62 (Fig. 1e–g, yellow arrowheads), as well as the autophagic membrane marker LC3B (Fig. 1h–j, white arrows). We confirmed through immunofluorescence that endogenous ubiquitin, p62, and LC3B also resided on ROS-stressed peroxisomes (Supplementary Fig. 5c–h). All of the above indicated to us that pexophagy was initiated to drive peroxisome turnover^22^.

**Fig. 2:**
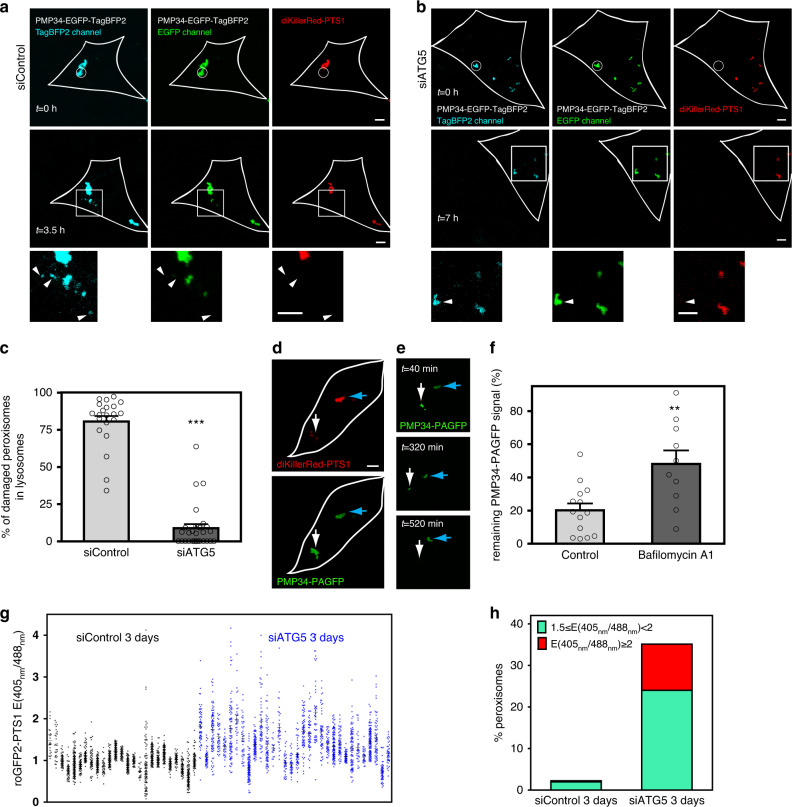
ROS-stressed peroxisomes are removed by macroautophagy. **a** Peroxisomes within the white circular region in a NIH3T3 cell expressing PMP34-EGFP-TagBFP2 and diKillerRed-PTS1 were illuminated with 559 nm light (top panels). Middle panels: the same cell 3.5 h after illumination. Bottom panels: magnified view of the white square region in the middle panels. The white arrows indicate the eventual loss of EGFP fluorescence on damaged peroxisomes. **b** The experiment in **a** was similarly carried out on a NIH3T3 cell transfected with a siRNA targeting ATG5. **c** The percentage of ROS-stressed peroxisomes that entered lysosomes (TagBFP2^+^ EGFP^−^) 7 h, following 559 nm illumination (siControl, *n* = 21 cells; siATG5, *n* = 28 cells; ***P = 1.41E−20, one-tailed *t* test). All error bars represent the mean + SEM. **d**–**f** NIH3T3 cells expressing diKillerRed-PTS1 and PMP34-PAGFP were 405 nm light illuminated to activate the whole-cell PMP34-PAGFP fluorescence. The eventual loss of PMP34-PAGFP signal was taken to indicate the delivery of peroxisomes into lysosomes for turnover. **d**, **e** ROS-stressed (559 nm light, white arrow) peroxisomes turned over much faster than non-stressed ones (blue arrow, *n* = 10 cells). **d** A NIH3T3 cell right after 559 nm light illumination. **e** The cell in **d** 40, 320, and 520 min after 559 nm light illumination. **f** The percentage of PMP34-PAGFP intensities remaining from ROS-stressed peroxisomes 8 h following 559 nm illumination were calculated. Bafilomycin A1 treatment delayed cellular turnover of ROS-stressed peroxisomes (*n* = 14 and 10 cells). All error bars represent the mean + SEM. **P = 0.0014 (one-tailed *t* test). All scale bars, 5 µm. **g**, **h** The redox sensor roGFP2-PTS1 was used to assay if ATG5 depletion results in cellular accumulation of ROS-stressed peroxisomes. **g** roGFP2-PTS1 emission ratios between 405 and 488 nm excitation. Each dot represents one peroxisome, and a column denotes all peroxisomes within a single cell. The ratios in ATG5-depleted cells vs. those in control cells 72 h after depletion are shown. **h** Graph tabulating the percentage of peroxisomes in **g** with roGFP2-PTS1 emission ratios between 1.5–2 (green) and ≧2 (red). siControl: *n* = 25 cells; siATG5: *n* = 32 cells. All scale bars, 5 µm. Source data are provided as a Source data file.

We then utilized three additional means to further validate that pexophagy was triggered. First, we monitored the delivery of the peroxisomal membrane protein PMP34 into lysosomes (following 559 nm illumination) using PMP34-EGFP^-^TagBFP2. Much like the frequently used EGFP-mRFP reporter constructs^23^, EGFP-TagBFP2 can be used to monitor lysosome delivery because of TagBFP2’s low pKa (2.7)^24^. We observed that PMP34-EGFP-TagBFP2 on ROS-stressed peroxisomes (Fig. 2a, top panels) eventually lost their EGFP fluorescence over time, while retaining their TagBFP2 signals (Fig. 2a, white arrowheads, bottom panels), suggesting their entrance into the acidic lysosomal environment. Cellular depletion of Atg5 (Supplementary Fig. 3a), on the other hand, blocked this PMP34 signal change (Fig. 2b, c). Second, using PMP34 as a marker, we found that ROS-stressed peroxisomes in time became colocalized with the lysosomal marker Lamp1 (Supplementary Fig. 1). Third, we monitored the disappearance of ROS-stressed peroxisomes using a photoactivatable peroxisome marker PMP34-PAGFP. Comparing 559 nm illuminated and non-illuminated peroxisomes marked with PMP34-PAGFP revealed that ROS-stressed peroxisomes disappeared at a much faster rate (Fig. 2d, e; illuminated: white arrows vs. non-illuminated: blue arrows). As disappearance can result from degradation, as well as peroxisomes moving out of the focal plane during long-term time-lapse imaging, we utilized the average PAGFP intensity ratios between illuminated and non-illuminated peroxisomes to better indicate degradation (as both populations can move perpendicular to the imaging plane). Using this, we found that degradation rate of ROS-stressed peroxisomes was significantly slowed by Bafilomycin A1 treatments (Fig. 2f). The above evidences confirmed that pexophagy was indeed the peroxisome quality control pathway observed^22^.

That our observed pexophagy can quality control peroxisomes suggests that a general block in autophagy would lead to cellular accumulation of dysfunctional peroxisomes. Indeed, we found that while most peroxisomes had similar redox states in control cells, Atg5-depleted cells possessed increased number of oxidatively stressed peroxisomes (Fig. 2g, h). As this experiment was done in the absence of any light-induced ROS production, it further suggests that peroxisomes stressed by endogenous ROS can also be turned over by pexophagy.

### Stub1 translocates onto ROS-stressed peroxisomes to mediate pexophagy

Knowing that cells can target individually ROS-stressed peroxisomes to the pexophagy pathway, we next asked if any specific ubiquitin E3 ligases carry out this task. It is known that ubiquitin E3 ligases plays instrumental roles in defining organelle quality control pathways. For example, it has been found that Parkin mediates ubiquitination of dysfunctional mitochondria to drive efficient mitophagy^25^. It has also been proposed that SCFFBXO27 ubiquitinates glycoproteins exposed on ruptured lysosomes to enhance lysophagy^26^.

**Fig. 3:**
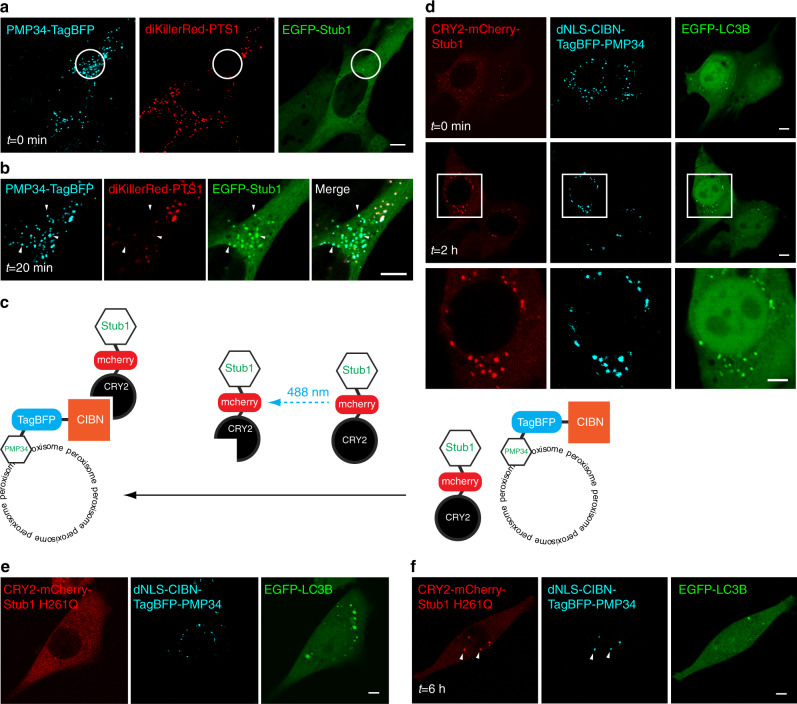
Peroxisomal Stub1 drives pexophagy. **a** Peroxisomes within the white circular region in a NIH3T3 cell expressing diKillerRed-PTS1, PMP34-TagBFP, and EGFP-Stub1 were 559 nm illuminated, leading to the immediate loss of their diKillerRed-PTS1 fluorescence. **b** EGFP-Stub1 specifically accumulated on 559 nm illuminated peroxisomes in the cell in **a** (white arrowheads, 20 min after 559 nm illumination, *n* = 16 cells). **c** Scheme to target Stub1 onto non-stressed peroxisomes. Upon 488 nm light illumination, CRY2-mCherry-Stub1 recruits to the surface of dNLS-CIBN-TagBFP-PMP34-labeled peroxisomes through CRY2/CIBN dimerization. **d** A NIH3T3 cell expressing CRY2-mCherry-Stub1, EGFP-LC3B, and dNLS-CIBN-TagBFP-PMP34 was whole-cell illuminated with 488 nm (19 μW, 2 min), leading to CRY2-mCherry-Stub1 recruitment onto all peroxisomes. This led to the EGFP-LC3B accumulation on all peroxisomes (top row: before 488 nm illumination; middle row: 2 h after illumination; bottom row: magnified view of the white square region in the middle row, *n* = 5 cells). **e**, **f** As in **d**, but with CRY2-mCherry-Stub1 H261Q. In this case, EGFP-LC3B did not accumulate on peroxisomes either before (**e**) or after illumination (**f**, white arrowheads, up to 6 h after 488 nm illumination, *n* = 12 cells). All scale bars: 5 μm.

We elicited peroxisomal ROS generation, and through time-lapse imaging found that an ubiquitin E3 ligase reporter construct EGFP-Stub1 translocated specifically to the ROS-stressed peroxisomes (Fig. 3a, b; white arrowheads). EGFP-Stub1 did not translocate onto light-stressed mitochondria (using KillerRed targeted into mitochondria in HeLa cells expressing Parkin, a model frequently used in studying Parkin-mediated mitophagy; Supplementary Fig. 2a, b), indicating that Stub1 possesses certain specificity toward peroxisomes. We also confirmed using immunofluorescence that endogenous Stub1 targeted to ROS-stressed peroxisomes (Supplementary Fig. 5a, b). Treating cells with the catalase inhibitor 3-amino-1, 2, 4-triazole (3-AT) to promote endogenous peroxisomal ROS production also led to the Stub1 recruitment onto peroxisomes (Supplementary Fig. 6, white arrows). To determine whether Stub1 can signal for the observed pexophagy, we probed if targeting Stub1 onto functional peroxisomes was sufficient to elicit their turnover by autophagy. We achieved this by utilizing a light-inducible dimerization system CRY2/CIBN^27^. CRY2 is a light-sensitive protein domain derived from *Arabidopsis* that, upon illumination, changes its conformation and become bound to CIBN. By placing CIBN onto peroxisomes through its fusion with PMP34, it became possible to target CRY2-mcherry-Stub1 onto functional peroxisomes through blue light-induced CRY2/CIBN dimerization (Fig. 3c)^27^. Indeed, 488 nm illumination resulted in CRY2-mcherry-Stub1 recruitment to peroxisomes (Fig. 3d), as well as the successful induction of pexophagy, as evidenced by the accumulation of EGFP-LC3B at the same foci. Targeting CRY2-mcherry-Stub1 to non-stressed peroxisomes also led to the peroxisomal ubiquitination (Supplementary Fig. 4a–c), another indication that the same ubiquitin-dependent pexophagy (similar to that observed with ROS-stressed peroxisomes) was induced. Targeting CRY2-mcherry to peroxisomes alone did not result in pexophagy (Supplementary Fig. 4d, e), indicating that our observed effect with CRY2-mcherry-Stub1 was not a result of 488 nm illumination, the CRY2/CIBN system, or the fluorescent protein tags. Targeting the known ligase-dead Stub1 (Stub1 H261Q) onto non-stressed peroxisomes also did not trigger pexophagy (Fig. 3e, f)^28^. Taken together, these results suggest that peroxisome-localized Stub1 is sufficient to initiate ubiquitin-dependent pexophagy, and that this Stub1 function relies on its ubiquitin ligase activities.

**Fig. 4:**
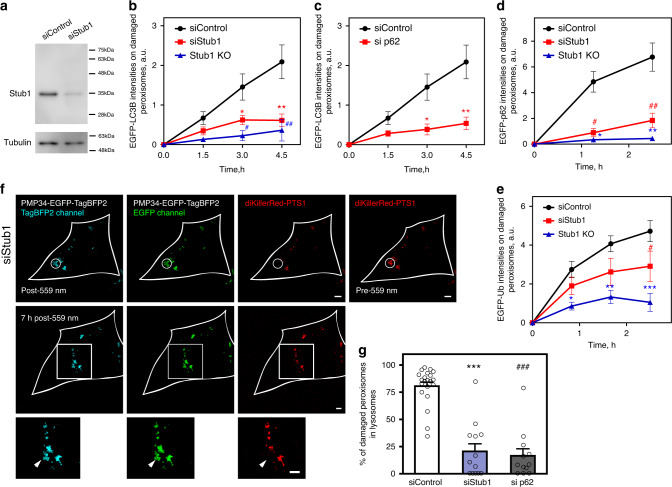
Stub1 depletion blocks pexophagy. **a** Stub1 depletion efficiency (siRNA) in our experiments. **b** Depleting Stub1 in NIH3T3 cells reduced EGFP-LC3B accumulation on ROS-stressed peroxisomes (quantified LC3B accumulation on ROS-stressed peroxisomes using the experimental setup in Fig. 1h–j; siControl, *n* = 10 cells; siStub1, *n* = 16 cells; Stub1 KO, *n* = 8 cells). *P = 0.0051, **P = 0.0004, ^#^*P* = 0.0023, and ^#^^#^*P* = 0.0019 (one-tailed *t* test). **c** Depleting p62 similarly reduced EGFP-LC3B accumulation on ROS-stressed peroxisomes (siControl, *n* = 10 cells; si p62, *n* = 10 cells). *P = 0.0039 and **P = 0.0007 (one-tailed *t* test). **d** Depleting Stub1 in NIH3T3 cells reduced EGFP-p62 accumulation on ROS-stressed peroxisomes (siControl, *n* = 6 cells; siStub1, *n* = 12 cells, Stub1 KO, *n* = 11 cells). *P = 8.77E−07, **P = 4.90E−07, ^#^*P* = 2.33E−05, and ^#^^#^*P* = 0.0002 (one-tailed *t* test). **e** Depleting Stub1 in NIH3T3 cells reduced EGFP-Ub accumulation on the ROS-stressed peroxisomes (siControl, *n* = 10 cells; siStub1, *n* = 11 cells, and Stub1 KO, *n* = 10 cells). *P = 0.0004 **P = 3.29E−05, ***P = 3.51E-05, and ^#^P = 0.0393 (one-tailed *t* test). All error bars in **b**–**e** represent mean ± SEM. **f** The peroxisomes within the white circular region in a NIH3T3 cells expressing PMP34-EGFP-TagBFP2 and diKillerRed-PTS1 were illuminated with 559 nm (top panels). Depletion of Stub1 reduced the rate of EGFP signals loss from ROS-stressed peroxisomes (middle panels: 7 h after 559 nm illumination; bottom panels: magnified view of the white square region in the middle panels). **g** The percentage of peroxisomes that entered lysosomes (TagBFP2^+^ EGFP^−^) 7 h following 559 nm illumination (siControl, *n* = 21 cells; siStub1, *n* = 13 cells, and si p62, *n* = 12 cells, ***P = 9.63E−10, ^#^^#^^#^*P* = 9.04E−11, one-tailed *t* test). All error bars represent mean + SEM. All scale bars: 5 μm. Source data are provided as a Source data file.

Is Stub1 necessary for pexophagy of ROS-stressed peroxisomes? Both depleting and knocking out cellular Stub1 (Fig. 4a, Supplementary Fig. 3b, Supplementary Table 1 and “Methods”) abolished ubiquitin, p62, as well as EGFP-LC3B accumulation on ROS-stressed peroxisomes (Fig. 4b, d, e and Supplementary Fig. 7a–c). Depleting p62 (Supplementary Fig. 3c) also diminished EGFP-LC3B accumulation on ROS-stressed peroxisomes (Fig. 4c). Depleting either Stub1 or p62 also blocked the delivery of ROS-stressed peroxisomes into lysosomes (monitored using PMP34-EGFP-TagBFP2; Fig. 4f, g). We found that the ligase-dead Stub1 H261Q, when expressed, blocked turnover of ROS-stressed peroxisomes, and led to their long-term accumulation within the cytoplasm (Supplementary Fig. 7d–f; white arrowheads). These confirmed the key roles Stub1 plays in pexophagy of ROS-stressed peroxisomes.

We further probed if Stub1-mediated pexophagy (observed within NIH3T3 cells) also occurs in human cells. To this end, we monitored the fate of ROS-stressed peroxisomes in HeLa and U2OS cells. We found that we could similarly observe the accumulation of Stub1, ubiquitin, p62, and LC3B on ROS-stressed peroxisomes in human cells (Supplementary Fig. 8).

### Heat shock protein 70s mediate Stub1 translocation onto ROS-stressed peroxisomes

**Fig. 5:**
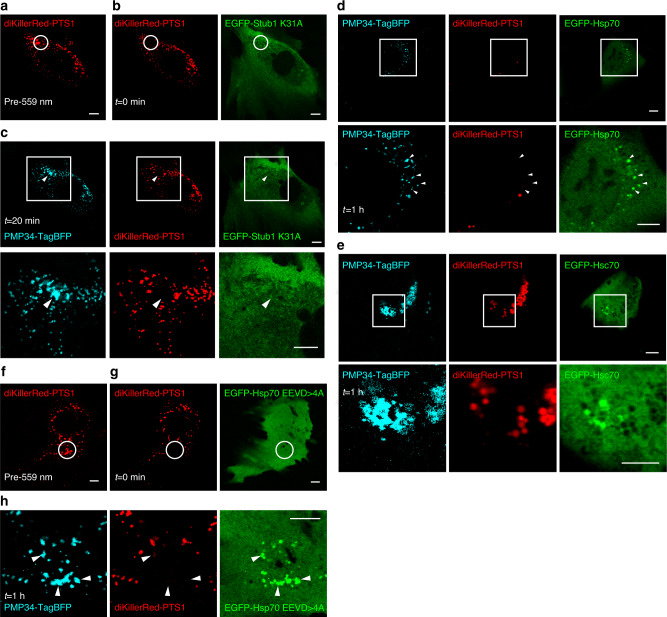
Hsc70/Hsp70 mediates Stub1 translocation onto ROS-stressed peroxisomes. A NIH3T3 cell expressing diKillerRed-PTS1, PMP34-TagBFP, and EGFP-Stub1 K31A was 559 nm illuminated in the white circular region (**a**), leading to the immediate loss in diKillerRed-PTS1 fluorescence (**b**). EGFP-Stub1 K31A did not accumulate on the ROS-stressed peroxisomes (**c**, top row, white arrowheads; bottom row: magnified view of the white square region in the top row, *n* = 4 cells). **d** Peroxisomes in a NIH3T3 cell expressing diKillerRed-PTS1, PMP34-TagBFP, and EGFP-Hsp70 were ROS stressed through 559 nm light illumination. Later EGFP-Hsp70 accumulated on ROS-stressed peroxisomes (white arrowheads; bottom panels represent magnified views of the white square region in top panels; 1 h after illumination shown, *n* = 12 cells). **e** As in **d**, but with EGFP-Hsc70 expression. Hsc70 similarly accumulated on ROS-stressed peroxisomes (bottom panels represent magnified views of the top panels; 1 h after illumination shown; *n* = 7 cells). **f** A NIH3T3 cell expressing diKillerRed-PTS1, PMP34-TagBFP, and EGFP-Hsp70 EEVD > 4 A was 559 nm illuminated in the white circular region, leading to the immediate loss in diKillerRed-PTS1 fluorescence (**g**). **h** Later EGFP-Hsp70 EEVD > 4 A accumulated on ROS-stressed peroxisomes (white arrowheads; 1 h after illumination shown; *n* = 4 cells). All scale bars: 5 μm.

Stub1 is known to rely on heat shock protein 70 s (Hsp70 and Hsc70) for the recognition and ubiquitination of a variety of substrates^29^. We found that Stub1 K31A, a mutant defective in its ability to bind Hsp70/Hsc70, could not localize onto ROS-stressed peroxisomes (Fig. 5a–c, white arrowheads), raising the possibility that Hsp70/Hsc70 help recruit Stub1 to ROS-stressed peroxisomes^30^. Using EGFP-tagged reporter constructs for Hsp70, Hsc70, as well as for their co-chaperone Hsp40, it was determined that all three, like Stub1, translocate onto ROS-stressed peroxisomes (Hsp70: Fig. 5d; Hsc70: Fig. 5e; Hsp40: Supplementary Fig. 9a–c). Interestingly, mutation that abolishes Hsp70 binding to Stub1 did not block Hsp70 targeting to ROS-stressed peroxisomes (Fig. 5f–h). On the other hand, the mutation blocked Stub1 translocation (Supplementary Fig. 9m). This demonstrates Hsp70’s ability to recognize dysfunctional peroxisomes^31,32^.

**Fig. 6:**
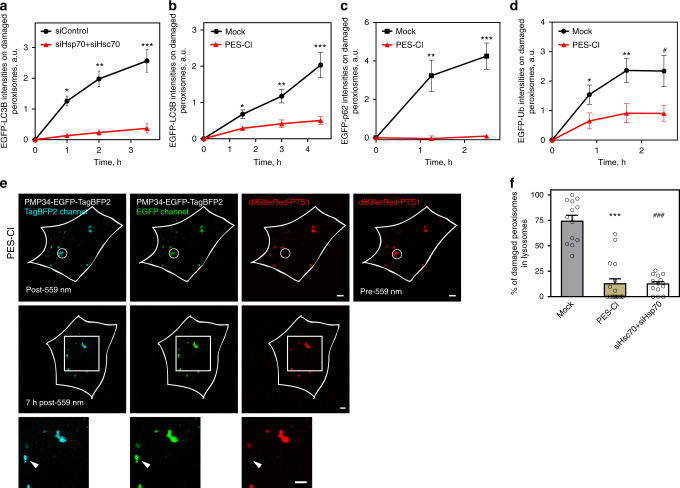
Inhibiting Hsc70/Hsp70 blocks pexophagy. **a** Quantifying LC3B accumulation on ROS-stressed peroxisomes (as in Fig. 1h–j). Depletion of cellular Hsc70 and Hsp70 delayed LC3B appearance on ROS-stressed peroxisomes (*x*-axis represents time after 559 nm illumination; siControl, *n* = 8 cells; siHsp70 + siHsc70, *n* = 18 cells). *P = 4.25E−09, **P = 8.32E−09, ***P = 3.22E−07 (one-tailed *t* test). **b** Hsp70/Hsc70 inhibition on LC3B accumulation. Treating cells with PES-Cl delayed LC3B appearance on ROS-stressed peroxisomes (Mock, *n* = 10 cells; PES-Cl, *n* = 14 cells). *P = 0.0011, **P = 0.0004, and ***P = 3.89E−05 (one-tailed *t* test). **c** Inhibiting Hsc70/Hsp70 with PES-Cl delayed EGFP-p62 accumulation on ROS-stressed peroxisomes (Mock, *n* = 9 cells; PES-Cl, *n* = 10 cells). **P = 0.0018 and ***P = 0.0001 (one-tailed *t* test). **d** PES-Cl treatment delayed EGFP-Ub accumulation on ROS-stressed peroxisomes (Mock, *n* = 11 cells; PES-Cl, *n* = 15 cells). *P = 0.0227, **P = 0.0046, and ^#^*P* = 0.0071 (one-tailed *t* test). All error bars in **a**–**d** represent mean ± SEM. **e** Same experimental setup as Fig. 2a, b, but with PES-Cl treatment. Peroxisomes within the white circular region in a NIH3T3 cell expressing PMP34-EGFP-TagBFP2 and diKillerRed-PTS1 were illuminated with 559 nm light (top panels). The EGFP and TagBFP2 signals of 559 nm illuminated peroxisomes were monitored 7 h after illumination (middle panels). The white arrows indicate that EGFP fluorescence persisted on ROS-stressed peroxisomes (bottom panels; magnified view of the white square region in middle panels). **f** The percentage of peroxisomes that entered lysosomes (TagBFP2^+^ EGFP^−^) 7 h following 559 nm illumination (Mock, *n* = 13 cells; PES-Cl, *n* = 17 cells; siHsc70 + siHsp70, *n* = 14 cells. ***P = 3.67E−09, ^#^^#^^#^*P* = 1.56E−08, one-tailed *t* test). All error bars represent the mean + SEM. All scale bars: 5 μm. Source data are provided as a Source data file.

We further validated that heat shock protein 70 s control Stub1-mediated pexophagy. Depleting cellular Hsp70/Hsc70 levels through the use of siRNA (Supplementary Fig. 3d, e), effectively blocked EGFP-LC3B accumulation on ROS-stressed peroxisomes (Fig. 6a). PES-Cl, a molecule that inhibits Hsp70/Hsc70 functions through binding to their substrate-binding domains, prevented Hsp70 (Supplementary Fig. 9d–f), Hsc70 (Supplementary Fig. 9g–i), and Stub1 (Supplementary Fig. 9j–l) from targeting to light-illuminated peroxisomes^33^. Treating cells with PES-Cl abolished ubiquitin, p62, as well as EGFP-LC3B accumulation on ROS-stressed peroxisomes (Fig. 6b–d). Depleting Hsp70/Hsc70, or treating cells with PES-Cl, also blocked the delivery of ROS-stressed peroxisomes into lysosomes (monitored using PMP34-EGFP-TagBFP2; Fig. 6e, f). These all support the notion that Hsp70/Hsc70 spots dysfunctional peroxisomes within the cytoplasm, and helps recruit Stub1 to trigger pexophagy to perform quality control.

### Depleting Stub1 or Hsp70/Hsc70 results in cellular accumulation of impaired peroxisomes

**Fig. 7:**
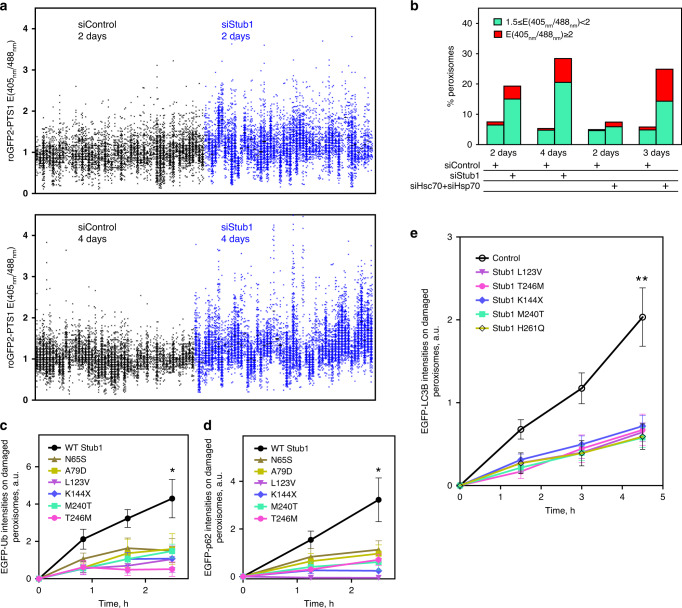
Ataxia-related Stub1 mutants affect peroxisomal ROS-mediated pexophagy. **a**, **b** We assayed if Stub1 depletion affected peroxisome quality using NIH3T3 cells expressing a peroxisomal redox sensor roGFP2-PTS1. **a** roGFP2-PTS1 emission ratios between 405 and 488 nm excitation. Each dot represents one peroxisome, and a column denotes all peroxisomes within a single cell. The ratios in Stub1-depleted cells vs. those in control cells are shown (top: 48 h after depletion; bottom: 96 h after depletion). **b** Graph tabulating the percentage of peroxisomes with roGFP2-PTS1 ratios between 1.5–2 (green) and ≧2 (red). **c**, **d** The effect of WT and Ataxia-related Stub1 mutant overexpression on ubiquitin and p62 recruitment onto ROS-stressed peroxisomes in Stub1 KO (NIH3T3) cells. Stub1 KO cells expressed EGFP-Ub (**c**) or EGFP-p62 (**d**), plus TagBFP-PMP34, diKillerRed-PTS1, and WT hStub1 or Ataxia-related Stub1 mutants tagged with TagBFP. **c** WT, *n* = 11 cells; N65S, *n* = 14 cells; A79D, *n* = 9 cells; L123V, *n* = 8 cells; K144X, *n* = 9 cells; M240T, *n* = 11 cells; T246M, *n* = 6 cells. Each mutant vs. WT Stub1: N65S, *P* = 0.0123; A79D, *P* = 0.0315; L123V, *P* = 0.0143; K144X, *P* = 0.0066; M240T, *P* = 0.0119; T246M, *P* = 0.0023 (one-tailed *t* test). **d** WT, *n* = 13 cells; N65S, *n* = 10 cells; A79D, *n* = 13 cells; L123V, *n* = 10 cells; K144X, *n* = 11 cells; M240T, *n* = 9 cells; T246M, *n* = 12 cells. Each mutant vs. WT Stub1: N65S, *P* = 0.0259; A79D, *P* = 0.0186; L123V, *P* = 0.0021; K144X, *P* = 0.0038; M240T, *P* = 0.0090; T246M, *P* = 0.0100 (one-tailed *t* test). **e** The effect of Ataxia-related Stub1 mutant expression on LC3B recruitment onto ROS-stressed peroxisomes in NIH3T3 cells expressing diKillerRed-PTS1, EGFP-LC3B, and PMP34-TagBFP. Control, *n* = 10 cells; L123V, *n* = 10 cells; K144X, *n* = 13 cells; M240T, *n* = 11 cells; T246M, *n* = 11 cells; H261Q, *n* = 10 cells. Each mutant vs. Control: L123V, *P* = 0.0016; K144X, *P* = 0.0023; M240T, *P* = 0.0012; T246M, *P* = 0.0012; H261Q, *P* = 0.0013 (one-tailed *t* test). All error bars in **c**–**e** represent the mean ± SEM. Source data are provided as a Source data file.

That heat shock protein 70 s and Stub1 can team up to quality control peroxisomes suggests that a lack in these molecules would lead to the cellular accumulation of dysfunctional peroxisomes. Again while most peroxisomes had similar redox state in control cells, Stub1-depleted cells possessed increased number of oxidatively stressed peroxisomes (Fig. 7a; in the absence of any light-induced ROS production). The fraction of oxidative peroxisomes increased as the Stub1 depletion duration lengthened (Fig. 7b). Simultaneous depletion of Hsp70/Hsc70 also showed the same effect (Supplementary Fig. 10 and Fig. 7b). This supports the notion that Stub1-mediated pexophagy can selectively target oxidatively stressed peroxisomes that spontaneously arise under normal culturing conditions to maintain peroxisome redox balance and homeostasis.

### Ataxia-related Stub1 mutants are defective in their ability to signal ubiquitin-dependent pexophagy

We next assayed whether cells harboring Stub1 mutants found in diseased patients displayed defective pexophagy. Several Stub1 mutations have been identified as the cause of autosomal recessive Ataxia syndromes^34–36^. Patients carrying Stub1 mutations displayed lower number of Purkinje cells and neurons in the granular layer of their cerebellum^37^. Using EGFP-reporter constructs for different Stub1 point mutants, we found that Stub1 N65S and Stub1 A79D were defective in their ability to target onto ROS-stressed peroxisomes in NIH3T3 cells (Table 1; assayed using the scheme in Fig. 1a). N65S/A79D mutants likely cannot effectively translocate onto ROS-stressed peroxisomes because of their weakened interactions with Hsp70s. N65 and A79 lie within the TRP domain of Stub1, the domain that mediates Stub1/Hsp70 binding^31^. Mutants carrying L123V, K144X, M240T, and T246M, on the other hand, could translocate onto ROS-stressed peroxisomes (Table 1). Expression of each of the mutants blocked ubiquitin, p62, and EGFP-LC3B accumulation on ROS-stressed peroxisomes (to the same degree as when expressing the ligase-dead Stub1 H261Q; Fig. 7c–e). These suggest that in Ataxia patients Stub1-mediated pexophagy will be defective. This will lead to the inadequate peroxisomal quality control, and may contribute to the development of Ataxia.

**Table 1:** Ataxia-related Stub1 mutants tested.

Disease-related Stub1 mutations	Localizes onto peroxisomes?
N65S	No
A79D	No
L123V	Yes
K144X	Yes
M240T	Yes
T246M	Yes

Right column denotes whether each mutant (EGFP-tagged) accumulated on ROS-stressed peroxisomes, when probed with experimental scheme used in Fig. 3a, b (N65S, *n* = 11 cells; A79D, *n* = 10 cells; L123V, *n* = 9 cells; K144X, *n* = 7 cells; M240T, *n* = 7 cells; T246M, *n* = 7 cells).

## Discussion

It was previously reported that cells treated with hydrogen peroxide will activate their pexophagy to reduce cellular peroxisome numbers^11^. Under those conditions, cellular Ataxia-telangiectasia mutated (ATM) translocates onto peroxisomes, and in turn drives PEX5 phosphorylation (Ser 141) and mono-ubiquitination (Lys 209) to mediate peroxisomal turnover by pexophagy. Given that this ATM-mediated action on peroxisomes is ROS-dependent, we wondered whether ATM would contribute to our observed Stub1-mediated pexophagy. We found that neither blocking PEX5 phosphorylation (by knocking down the endogenous PEX5 while at the same time expressing PEX5 S141A through plasmid transfection) nor preventing PEX5 mono-ubiquitination (by knocking down the endogenous PEX5, while at the same time expressing PEX5 K209R through plasmid transfection) affected EGFP-LC3 accumulation onto ROS-stressed (559 nm illuminated) peroxisomes (Supplementary Fig. 11a–c). Inhibiting cellular ATM activities through the use of KU-55933 also did not attenuate the accumulation of ubiquitin or Stub1 onto ROS-stressed peroxisomes (Supplementary Fig. 11d, e). Together these suggest that Stub1-mediated pexophagy can individually quality control peroxisomes without help from the previously reported ATM activities^11^.

To what extent does Stub1-mediated pexophagy quality control peroxisomes in vivo? It will be particular interesting to investigate the conditions and settings where this pathway is most important. Efforts to determine cellular and organismic phenotypes associated with a lack of Stub1-mediated pexophagy will also be very beneficial. It will also be important to further the mechanistic details of this pathway, to allow one to understand what additional genetic/molecular defects will result in impaired cellular peroxisomal quality control.

Several clinical reports indicate that patients with Ataxia phenotypes harbor defects in their peroxisomal genes^5,38^. This suggests that inadequate peroxisome function or quality may be a contributing factor to the development of Ataxia. In accordance with this idea, we have found that Stub1 mutants from Ataxia patients are defective in their ability to initiate Stub1-mediated pexophagy. It will therefore be of general interest in the future to thoroughly investigate the connections between defects in various peroxisome quality control pathways, and the development of Ataxia.

## Methods

### Plasmids

All primers used for plasmid construction are listed in Supplementary Table 2. EGFP-Stub1 was generated by inserting the mouse Stub1 gene (through PCR amplification of the total mouse kidney cDNA) into EGFP-C1. diKillerRed-PTS1 was made by appending the KillerRed tandem dimer (two KillerRed moieties spaced with a 22 amino acid linker HGTGSTGSGSSGTASSEDNNMA) with the PTS1 VKSKL. EGFP-Ub, EGFP-Hsc70, and EGFP-LC3B were obtained from Addgene (plasmids 11928, 19487, and 11546). Redox sensor roGFP2-PTS1 was generated by appending eroGFP (taken from Addgene plasmid 20131) with the amino acid sequence VKSKL, and cloned into the EGFP-C1. EGFP-Hsp70, EGFP-Hsp40, EGFP-hStub1, PMP34-TagBFP, and TagBFP2-hStub1 were constructed by obtaining the respective genes from the total cDNA from HeLa cell lysates through PCR, followed by their insertion into EGFP-C1 or TagBFP2-C (Evrogen FP171 with I174 A). The PMP34 gene from HeLa was also inserted into the PAGFP plasmid (Addgene plasmid 18697) to generate PMP34-PAGFP. EGFP-linked Stub1 mutants H261Q, N65S, A79D L123V, K144X, M240T, T246M, and EGFP-Hsp70 EEVD > 4A (last four amino acids of Hsp70 replaced by four alanine residues), were all created through PCR-based site-directed mutagenesis using designed primers. The construct dNLS-CIBN-TagBFP-PMP34 (NLS: nuclear localization signal) was generated in three steps. First, a TagBFP-PMP34 construct was made by inserting human PMP34 into TagBFP-C (Evrogen FP171). Then, CIBN-TagBFP-PMP34 was constructed by PCR amplification of CIBN (amino acids 1–170) from pmCherry-CIBN-CreC (Addgene plasmid 26889), followed by its insertion into TagBFP-PMP34. The linker sequence between CIBN and TagBFP is HGGGGSGGGGSGLAVAT. Lastly, NLS (amino acid sequence 93–107) of CIBN was mutated from KRKFDTETKDCNEKK to AAKFDTETKDCNEAA to obtain dNLS-CIBN-TagBFP-PMP34. CRY2-mCherry-Stub1 H261Q was constructed by inserting mouse Stub1 H261Q into CRY2-mCherry (Addgene plasmid 58368). PMP34-EGFP was generated by inserting PMP34 into EGFP-C1. TagBFP2 cut from TagBFP2-N (Evrogen FP172) was inserted into PMP34-EGFP to obtain PMP34-EGFP-TagBFP2. PEX5-Myc was generated by inserting the human PEX5 gene (through PCR amplification of the HeLa cDNA) into the NheI site of TagBFP2-C, followed by the addition of Myc tag and stop codon (all in front of the TagBFP2 gene). PEX5 mutants (S141A and K209R) were created through PCR-based site-directed mutagenesis using designed primers.

### siRNAs

We set up 5 × 10^4^ cells grown overnight on 35 mm Mattek glass bottom dishes, and used 50–125 pmol of the following siRNAs along with Lipofectamine 2000 (Thermo Fisher Scientific, 11668) for knockdown experiments: siHsc70 (GE Dharmacon siGENOME SMARTpool mouse HspA8, M-062625-01), siHsp70 (GE Dharmacon siGENOME SMARTpool mouse HspA1A, M-054644-01), siControl (GE Dharmacon siGENOME Nontargeting siRNA Pool, D-001206-13), siStub1 (GE Dharmacon siGENOME SMARTpool mouse Stub1, M-063143-01), si p62 (GE Dharmacon siGENOME SMARTpool mouse Sqstm1, M-047628-01), siATG5 (GE Dharmacon siGENOME SMARTpool mouse Atg5, M-064838-02), and siPEX5 (GE Dharmacon siGENOME SMARTpool mouse PEX5, M-063682-01).

### Cell culture conditions

NIH3T3 cells (ATCC, CRL-1658) were cultured in Dulbecco’s Modified Eagle Medium (Thermo Fisher Scientific, 11965) supplemented with 10% bovine serum (Thermo Fisher Scientific, 16170-060) and 1% penicillin/streptomycin (Thermo Fisher Scientific, 15140). The cells were maintained at 37 °C and 5% CO_2_. HeLa cell line (ATCC and CCL-2) and U2OS human osteosarcoma cell line (Bioresource Collection and Research Center, Hsinchu, Taiwan) were maintained at 37 °C, 5% CO_2_ in Dulbecco’s Modified Eagle Medium supplemented with 10% fetal bovine serum (Thermo Fisher Scientific, 10437) and 1% penicillin/streptomycin (Thermo Fisher Scientific, 15140). Lipofectamine 2000 (Thermo Fisher Scientific, 11668) was used for plasmid transfections based on the manufacturer’s protocol. PES-Cl (Calbiochem, 5.31067) treatments were done by supplementing 50 μM of the drug in cell culture medium 2 h before light-assisted peroxisome damage. Cellular catalase inhibition was done by treating cells with 3-AT (Sigma-Aldrich, A8056) at the concentration of 150 mM for 14 h. Cellular ATM kinase inhibition was done by treating cells with KU-55933 (Sigma-Aldrich, SML1109) at 500 nM 2 h before 559 nm light-assisted peroxisome damage.

### SDS–PAGE and western blot

NIH3T3 cells cultured on 3.5 cm dishes were placed on ice, washed with PBS, and overlaid with 0.15 ml of ice-cold lysis buffer (62.5 mM Tris, pH 6.8, 2% SDS, 10% glycerol, 2 mM PMSF, and protease inhibitor cocktail tablet (Sigma, s8830)). The adherent cells were scraped off the dish. The cell suspensions were gently transferred into precooled micro-centrifuges tube and sonicated at 4 °C. SDS–PAGE sample buffer supplemented with 2 mM DTT were added to the crude lysates and boiled at 95–100 °C for 5 min. The samples were then separated on a 10% SDS–polyacrylamide gel and transferred onto PVDF membranes. Membranes were probed using the following primary antibody (condition: 4 °C overnight): anti-Hsp70/HspA1A (R&D system, MAB1663 mouse monoclonal to Hsp70) diluted 1:3500 in 6% milk/PBS, anti-Hsc70/HspA8 (Abcam, ab2788 mouse monoclonal to Hsc70) diluted 1:3500 in 6% milk/PBS, anti-Stub1 (Abcam, ab134064 rabbit monoclonal to Stub1) diluted 1:2000 in 6% milk/PBS, anti-p62 (Abcam, ab56416 mouse monoclonal to Sqstm1) diluted 1:2000 in 6% milk/PBS, anti-ATG5 (GeneTex, GTX62601 rabbit monoclonal to ATG5) diluted 1:1250 in 6% milk/PBS, and anti-PEX5 (Novus Biologicals, NBP1-87185 rabbit polyclonal) diluted 1:3500 in 6% milk/PBS. Anti-tubulin (Abcam, ab6160 rat monoclonal (YL1/2) to tubulin) was used to detect cellular tubulin as loading controls. Chemiluminescence signals were imaged using a Fujifilm LAS-3000. Full scans of all the blots used in this work are shown in Supplementary Fig. 12.

### Live cell manipulation and imaging

NIH3T3 cells were imaged on an Olympus FV1000 confocal microscope (UPLSAPO 60XS2, N.A. 1.3, silicon oil objective). NIH3T3 cells were grown in phenol red-free medium for observation (Thermo Fisher Scientific, 31053; containing 10% bovine serum and 1% penicillin/streptomycin). Live NIH3T3 cells were maintained in a microscope stage-top incubator (LCI Chamlide IC) under 37 °C and 5% CO_2_ for manipulation and observation. To oxidatively stress peroxisomes, we point-scanned 50 μW 559 nm laser light through a selected 1 μm diameter circular region for a total of 50 s, using Olympus FV1000’s tornado scanning. The resulting images were analyzed and quantified through the combined use of ImageJ 1.48 v, Excel 2013, and GraphPad Prism 5.

### Quantifying peroxisomal redox potential

NIH3T3 cells were transfected with roGFP2-PTS1 plus either 125 pmol of siStub1 or nontargeting siRNA (or in the heat shock protein 70 depletion experiments 100 pmol nontargeting siRNA or 50 pmol siHspA8 plus 50 pmol siHspA1A) through the use of Lipofectamine 2000. Cells were then imaged on an Olympus FV3000 microscope (PLAPON 60XOSC2 N.A. 1.4 oil objective) 48–72 h after transfection. Redox sensor roGFP2-PTS1 fluorescence was imaged using both 405 and 488 nm excitation (collecting 505–550 nm emission). ROIs marking individual peroxisomes were obtained through thresholding the 488-nm excited image in ImageJ. ROIs containing less than five pixels were eliminated. By utilizing the remaining ROIs (each marking a peroxisome), we calculated the signal ratios between the 405-nm excited and the 488-nm excited fluorescence for every peroxisome within a cell (using the analyze particles and ROI manager functions in ImageJ). These 405 nm/488 nm ratios were taken to indicate the redox potential of individual peroxisomes.

### Quantification of peroxisomal EGFP-LC3B/EGFP-p62/EGFP-Ub following damage

NIH3T3 cells were transfected with diKillerRed-PTS1, PMP34-TagBFP, and EGFP-LC3B/EGFP-p62/EGFP-Ub. Peroxisomes were damaged by point-scanning a 50 μW 559 nm laser light through a 1 μm diameter circular area for a total of 50 s, using Olympus FV1000’s tornado scanning. The peroxisomes were identified through thresholding the images using PMP34-TagBFP signals in ImageJ. The average of green fluorescence on peroxisomes (before damage) were normalized as one and the fold intensity increase (Δ) were analyzed, using the analyze particle function in ImageJ.

### Monitoring turnover of damaged peroxisomes

We utilized PMP34-PAGFP to mark peroxisomes and tracked their fates following light-activated damage on the Olympus FV1000 confocal microscope. NIH3T3 cells were transfected with PMP34-PAGFP and diKillerRed-PTS1. The measurements were done in three steps: (1) photoactivate all PMP34-PAGFP within a focal plane in single cells through 405 nm illumination. (2) Oxidatively stress a portion of peroxisomes by point-scanning the 559 nm laser to generate ROS (mediated by diKillerRed-PTS1; damaged peroxisomes will become only green fluorescent due to KillerRed photobleaching, while normal peroxisomes will be both green and red). (3) Monitor the green fluorescence from activated PMP34-PAGFP over time. To quantify turnover, we normalized the total PMP34-PAGFP fluorescence on damaged peroxisomes to those before damage. The peroxisomes were identified through intensity thresholding PMP34-PAGFP fluorescence and quantified using the “analyze particles” function in ImageJ.

We used a tandem fluorescent-tagged peroxisome marker PMP34-EGFP-TagBFP2 to monitor delivery of ROS-stressed peroxisomes into acidic compartments (e.g., lysosomes). We transfected NIH3T3 cells with PMP34-EGFP-TagBFP2 and diKillerRed-PTS1 plus siRNA. Peroxisomes right after 559 nm illumination will become only blue and green fluorescent due to KillerRed photobleaching. Upon entering acidic compartment, damaged peroxisomes will then become only blue fluorescent due to EGFP quenching. Image analysis: the TagBFP2 and EGFP areas were identified through intensity thresholding and quantified using the “analyze particles” function in ImageJ.

### Targeting Cry2-mCherry-Stub1 to healthy peroxisomes

NIH3T3 cells were transfected with dNLS-CIBN-TagBFP2-PMP34, EGFP-LC3B, and CRY2-mCherry-Stub1 (or the CRY2-mCherry-Stub1 H261Q mutant). Sixteen hours following transfection, light-assisted CRY2-CIBN dimerization was induced by point-scanning 488 nm light through the entire cell for a total of 2 min on the Olympus FV1000.

### Immunofluorescence assay following LED illumination

NIH3T3 cells cultured on 3.5 cm dishes were transfected with diKillerRed-PTS1 and PMP34-TagBFP. One day later, all NIH3T3 cells on 3.5 cm dish were illuminated with 565 nm light, using LED (THORLABS, M565L2 500 mA) for 9 h. NIH3T3 cells were washed in PBS and then fixed using 4% paraformaldehyde in PBS pH 7.4 for 10 min at room temperature. Fixed cells were permeabilized with PBS containing 0.1% Triton X-100 for 10 min and blocked with 1% BSA in PBS for 1 h at room temperature. Cells were incubated with diluted antibody in 1% BSA in PBS in a humidified chamber overnight at 4 °C. Cells were washed three times in PBS for 5 min each and incubated with diluted secondary antibody in 1% BSA for 1 h at room temperature in the dark. After the removal of secondary antibody solution, NIH3T3 cells were three times PBS washed and imaged on an Olympus FV1000 confocal microscope. Cellular proteins were probed using the following primary antibodies (condition, 4 °C overnight): anti-Stub1 (Abcam, ab134064 rabbit monoclonal to Stub1) diluted 1:150 in 1% BSA/PBS, anti-Ub (Sigma-Aldrich, 04–263 mouse monoclonal to ubiquitinylated proteins) diluted 1:150 in 1% BSA/PBS, anti-p62 (Abcam, ab56416 mouse monoclonal to Sqstm1) diluted 1:150 in 1% BSA/PBS, and anti-LC3B (Novus Biologicals, NB100-2220 rabbit polyclonal to LC3B) diluted 1:250 in 1% BSA/PBS.

The green fluorescent secondary antibodies (Thermo Fisher Scientific, A11034) Alexa Fluor 488 goat anti-rabbit IgG (H + L) or (Thermo Fisher Scientific, A11029) Alexa Fluor 488 goat anti-mouse IgG (H + L) were diluted 1:1500 in 1% BSA/PBS for visualization.

### PEX5-Myc immunofluorescence

NIH3T3 cells were washed in PBS and then fixed using 4% paraformaldehyde in PBS pH 7.4 for 10 min at room temperature 3 days after PEX5-Myc transfection (wild type, S141A, or K209R). Fixed cells were permeabilized with −20 °C methanol for 15 min and blocked with 1% BSA in PBS for 1 h at room temperature. Cells were incubated with the diluted anti-Myc antibody (Cell Signaling, 2278 rabbit monoclonal; 1:600) in 1% BSA/PBS in a humidified chamber overnight at 4 °C. Cells were washed three times in PBS for 5 min each and incubated with Hoechst 33342 (1:1000 for nuclear counterstaining) and diluted secondary antibody (1:500, Thermo Fisher Scientific, A11034 Alexa Fluor 488 goat anti-rabbit IgG (H + L)) in 1% BSA/PBS. After the removal of secondary antibody solution, NIH3T3 cells were three times PBS washed and imaged on an Olympus FV1000 confocal microscope.

### Generating Stub1 knockout cells using CRISPR/Cas9

The Stub1 CRISPR/Cas9 sgRNA targeting sequences in mouse were designed using Benchling (Supplementary Table 1; https://benchling.com). Each target-specific sgRNA was in vitro transcribed from a designed DNA template (Supplementary Table 1), including a T7 promoter, a 20 nt targeting sequence (without PAM), and a published sgRNA scaffold^39^. The in vitro transcription products were purified by electrophoresis on a 10% polyacrylamide gel with 6 M urea. The gel pieces containing the denatured sgRNAs were grinded in 300 mM sodium acetate (pH 5.0), followed by sgRNA precipitation and purification. Purified/dried RNA pallets were dissolved into 20 mM HEPES (pH 7.5), 150 mM KCl, 10% glycerol, and 1 mM 2-mercaptoethanol, and refolded into a functional structure.

The Cas9 RNPs with sgRNAs targeting Stub1 were prepared by incubating the purified Cas9 protein^40^ (100 pmol) with sgRNA (120 pmol) at 1:1.2 molar ratio at 37 °C for 10 min. Nucleofection of NIH3T3 cells were performed on a Lonza SE Cell Line 4D-Nucleofector according to the manufacturer’s instructions (program EN-158). After nucleofection, cells were incubated with fresh medium at 37 °C for 48 h, followed by cell sorting using a BD FACSJazz automated cell sorter (Academia Sinica Flow Cytometry Core Facility) for single-cell isolation.

We confirmed Stub1 knockout by PCR. A 969 bp region of Stub1 (NIH3T3) containing the Cas9-RNP targeting site can be amplified by PCR (primers: forward 5′-GCCTCCAACT GCTTTTGAGAGAAAT-3′; reverse 5′-ACGTGCTTGTTTCTCAGCCATTAAT-3′). Cas9-induced site-specific double-strand DNA breaks will cause a 318 bp fragment deletion after nonhomologous end-joining repair. The PCR products from knockout clones will therefore be around 651 bp.

### Reporting summary

Further information on research design is available in the Nature Research Reporting Summary linked to this article.

## Supplementary information

Supplementary Information

Reporting Summary

## Data Availability

The data that support the findings of this study are available from the corresponding author upon reasonable request. Source data are provided with this paper.

## Source data

Source Data

## References

[1] Purdue PE, Lazarow PB (2001). Peroxisome biogenesis. Annu. Rev. Cell Dev. Biol..

[2] Reuber BE (1997). Mutations in PEX1 are the most common cause of peroxisome biogenesis disorders. Nat. Genet.

[3] Smith JJ, Aitchison JD (2013). Peroxisomes take shape. Nat. Rev. Mol. Cell Biol..

[4] Weller S, Gould SJ, Valle D (2003). Peroxisome biogenesis disorders. Annu. Rev. Genomics Hum. Genet.

[5] De Munter S, Verheijden S, Regal L, Baes M (2015). Peroxisomal disorders: a review on cerebellar pathologies. Brain Pathol..

[6] Wanders RJ, Waterham HR (2006). Peroxisomal disorders: the single peroxisomal enzyme deficiencies. Biochim. Biophys. Acta.

[7] Fransen M, Nordgren M, Wang B, Apanasets O (2012). Role of peroxisomes in ROS/RNS-metabolism: implications for human disease. Biochim. Biophys. Acta.

[8] Schrader M, Fahimi HD (2006). Peroxisomes and oxidative stress. Biochim. Biophys. Acta.

[9] Dunn WA (2005). Pexophagy: the selective autophagy of peroxisomes. Autophagy.

[10] Okamoto K (2014). Organellophagy: eliminating cellular building blocks via selective autophagy. J. Cell Biol..

[11] Zhang J (2015). ATM functions at the peroxisome to induce pexophagy in response to ROS. Nat. Cell Biol..

[12] Manjithaya R, Nazarko TY, Farre JC, Subramani S (2010). Molecular mechanism and physiological role of pexophagy. FEBS Lett..

[13] Iwata J (2006). Excess peroxisomes are degraded by autophagic machinery in mammals. J. Biol. Chem..

[14] Kim PK, Hailey DW, Mullen RT, Lippincott-Schwartz J (2008). Ubiquitin signals autophagic degradation of cytosolic proteins and peroxisomes. Proc. Natl Acad. Sci. USA.

[15] Yang JY, Yang WY (2011). Spatiotemporally controlled initiation of Parkin-mediated mitophagy within single cells. Autophagy.

[16] Hung YH, Chen LM, Yang JY, Yang WY (2013). Spatiotemporally controlled induction of autophagy-mediated lysosome turnover. Nat. Commun..

[17] Fransen M, Brees C (2017). KillerRed as a tool to study the cellular responses to peroxisome-derived oxidative stress. Methods Mol. Biol..

[18] Yang JY, Yang WY (2013). Bit-by-bit autophagic removal of parkin-labelled mitochondria. Nat. Commun..

[19] Bulina ME (2006). A genetically encoded photosensitizer. Nat. Biotechnol..

[20] Gould SG, Keller GA, Subramani S (1987). Identification of a peroxisomal targeting signal at the carboxy terminus of firefly luciferase. J. Cell Biol..

[21] Lismont C, Walton PA, Fransen M (2017). Quantitative monitoring of subcellular redox dynamics in living mammalian cells using RoGFP2-based probes. Methods Mol. Biol..

[22] Klionsky DJ (2016). Guidelines for the use and interpretation of assays for 803 monitoring autophagyy (3rd edition). Autophagy.

[23] Kimura S, Noda T, Yoshimori T (2007). Dissection of the autophagosome maturation process by a novel reporter protein, tandem fluorescent-tagged LC3. Autophagy.

[24] Subach OM, Cranfill PJ, Davidson MW, Verkhusha VV (2011). An enhanced monomeric blue fluorescent protein with the high chemical stability of the chromophore. PLoS ONE.

[25] Pickrell AM, Youle RJ (2015). The roles of PINK1, parkin, and mitochondrial fidelity in Parkinson’s disease. Neuron.

[26] Yoshida Y (2017). Ubiquitination of exposed glycoproteins by SCF(FBXO27) directs damaged lysosomes for autophagy. Proc. Natl Acad. Sci. USA.

[27] Kennedy MJ (2010). Rapid blue-light-mediated induction of protein interactions in living cells. Nat. Methods.

[28] Hatakeyama S, Yada M, Matsumoto M, Ishida N, Nakayama KI (2001). U box proteins as a new family of ubiquitin-protein ligases. J. Biol. Chem..

[29] Fernandez-Fernandez MR, Gragera M, Ochoa-Ibarrola L, Quintana-Gallardo L, Valpuesta JM (2017). Hsp70 - a master regulator in protein degradation. FEBS Lett..

[30] Xu W (2002). Chaperone-dependent E3 ubiquitin ligase CHIP mediates a degradative pathway for c-ErbB2/Neu. Proc. Natl Acad. Sci. USA.

[31] Ballinger CA (1999). Identification of CHIP, a novel tetratricopeptide repeat-containing protein that interacts with heat shock proteins and negatively regulates chaperone functions. Mol. Cell Biol..

[32] Wu SJ, Liu FH, Hu SM, Wang C (2001). Different combinations of the heat-shock cognate protein 70 (hsc70) C-terminal functional groups are utilized to interact with distinct tetratricopeptide repeat-containing proteins. Biochem. J..

[33] Balaburski GM (2013). A modified HSP70 inhibitor shows broad activity as an anticancer agent. Mol. Cancer Res..

[34] Heimdal K (2014). STUB1 mutations in autosomal recessive ataxias - evidence for mutation-specific clinical heterogeneity. Orphanet J. Rare Dis..

[35] Shi Y (2013). Identification of CHIP as a novel causative gene for autosomal recessive cerebellar ataxia. PLoS ONE.

[36] Synofzik M (2014). Phenotype and frequency of STUB1 mutations: next-generation screenings in Caucasian ataxia and spastic paraplegia cohorts. Orphanet J. Rare Dis..

[37] Bettencourt C (2015). Clinical and neuropathological features of spastic ataxia in a Spanish family with novel compound heterozygous mutations in STUB1. Cerebellum.

[38] Vilarinho S (2016). ACOX2 deficiency: a disorder of bile acid synthesis with transaminase elevation, liver fibrosis, ataxia, and cognitive impairment. Proc. Natl Acad. Sci. USA.

[39] Chen B (2013). Dynamic imaging of genomic loci in living human cells by an optimized CRISPR/Cas system. Cell.

[40] Lingeman E, Jeans C, Corn JE (2017). Production of purified CasRNPs for efficacious genome editing. Curr. Protoc. Mol. Biol..

