# Supplementary material for: Hsc70/Stub1 promotes the removal of individual oxidatively stressed peroxisomes
Source: Nat Commun. 2020 Oct 19;11:5267. doi: 10.1038/s41467-020-18942-3 (PMC7573593; doi:10.1038/s41467-020-18942-3)
Supplement: Supplementary file 1 — Supplementary Information [file 41467_2020_18942_MOESM1_ESM.pdf]

Supplementary information

Hsc70/Stub1 promotes the removal of individual oxidatively stressed peroxisomes

Chen et al.

Table of Contents:

-Supplementary Figures 1-11
-Supplementary Tables 1-2

Supplementary Figures

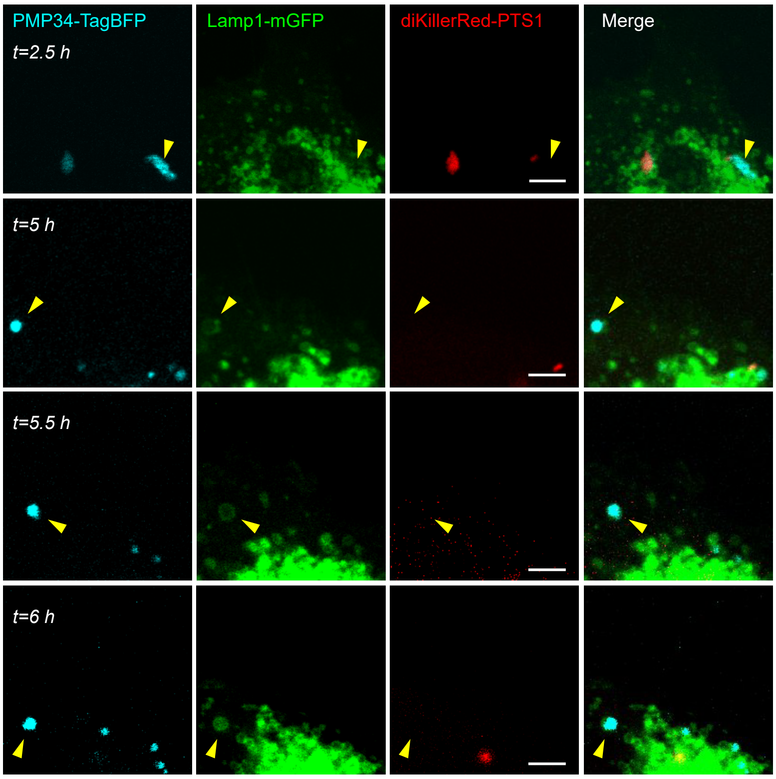

Supplementary Figure 1: Delivery of ROS-stressed peroxisomes into lysosomes.

Specific peroxisomes (yellow arrowhead) within a NIH3T3 cell expressing Lamp1-mGFP, PMP34-TagBFP, and diKillerRed-PTS1, were 559 nm illuminated. Illuminated peroxisomes eventually colocalized with Lamp1-mGFP (n=3 cells). All scale bars: 5 µm.

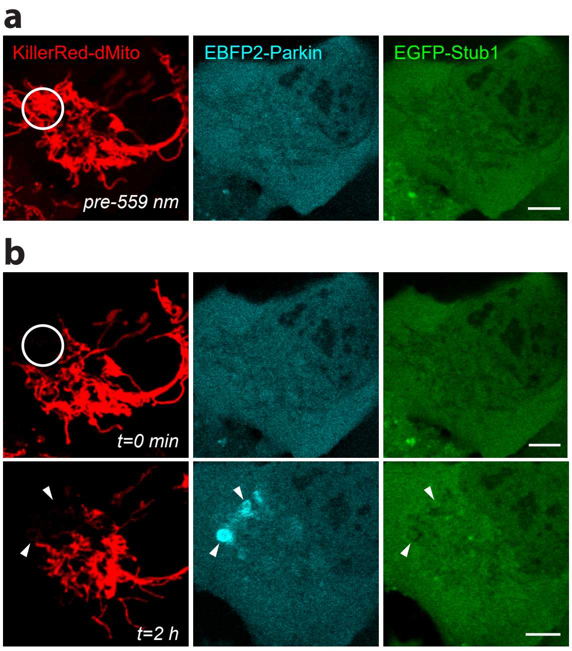

Supplementary Figure 2: Stub1 does not translocate onto damaged mitochondria.

a A HeLa cell expressing KillerRed-dMito, EGFP-Stub1 and EBFP2-Parkin. **b** Mitochondria were ROS-stressed through 559 nm illumination at the white circular region, leading to the immediate loss in KillerRed-dMito fluorescence (top panels). Two hours following illumination, EBFP2-Parkin accumulated on ROS-stressed mitochondria (white arrowheads, bottom panels). EGFP-Stub1, on the other hand, did not (n=3 cells). All scale bars: 5 μm.

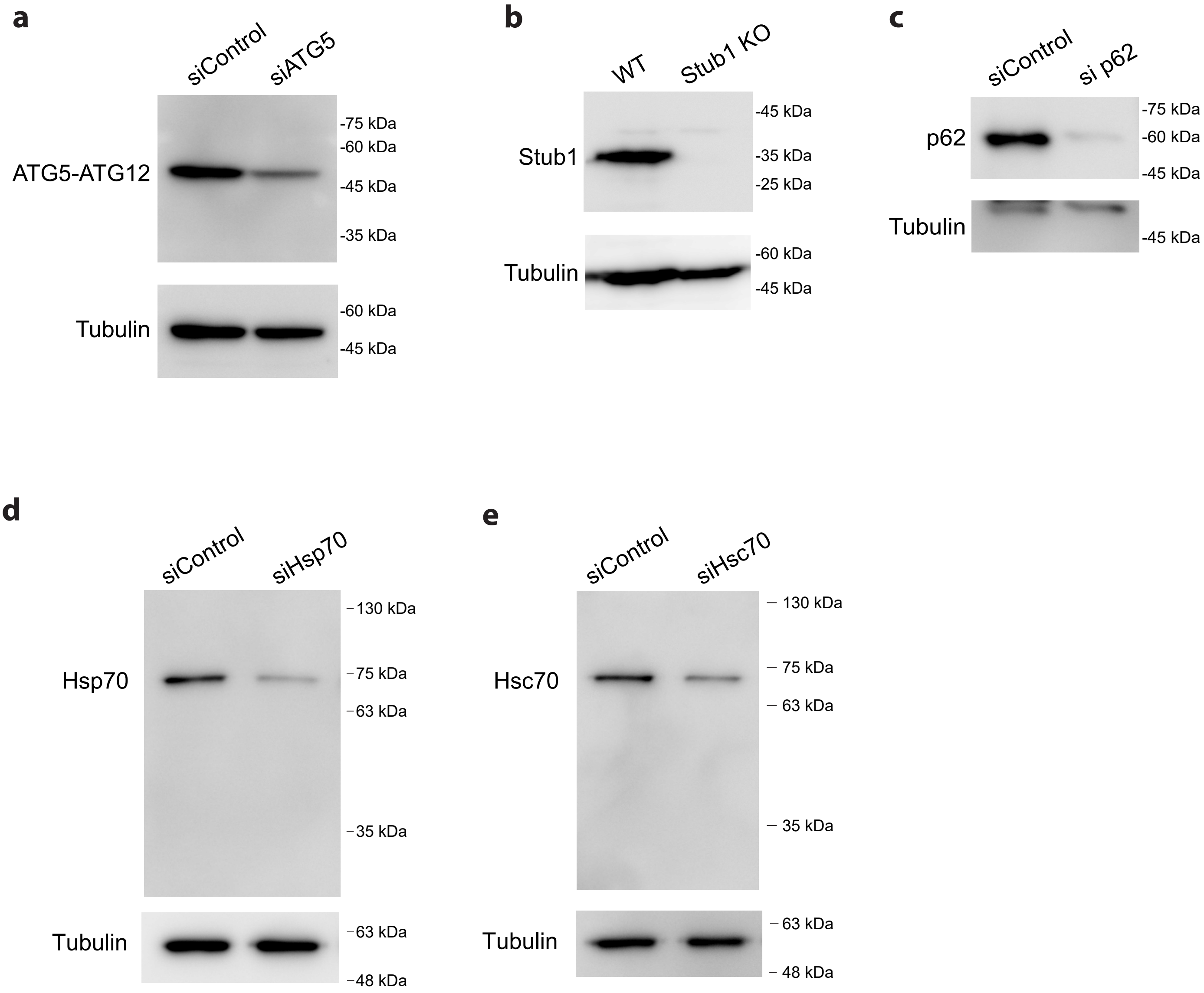

Supplementary Figure 3: siRNA knockdowns efficiencies.

a Efficiency of the ATG5 siRNA utilized in this paper (3 days after transfection). **b** Successful CRISPR/Cas9 mediated Stub1 knockout (Stub1 KO) in NIH3T3 cells. **c** Efficiency of the p62 siRNA utilized in this paper (3 days after transfection). **d-e** Efficiencies of the Hsp70 and Hsc70 siRNAs utilized in this paper. Source data are provided as a Source Data file.

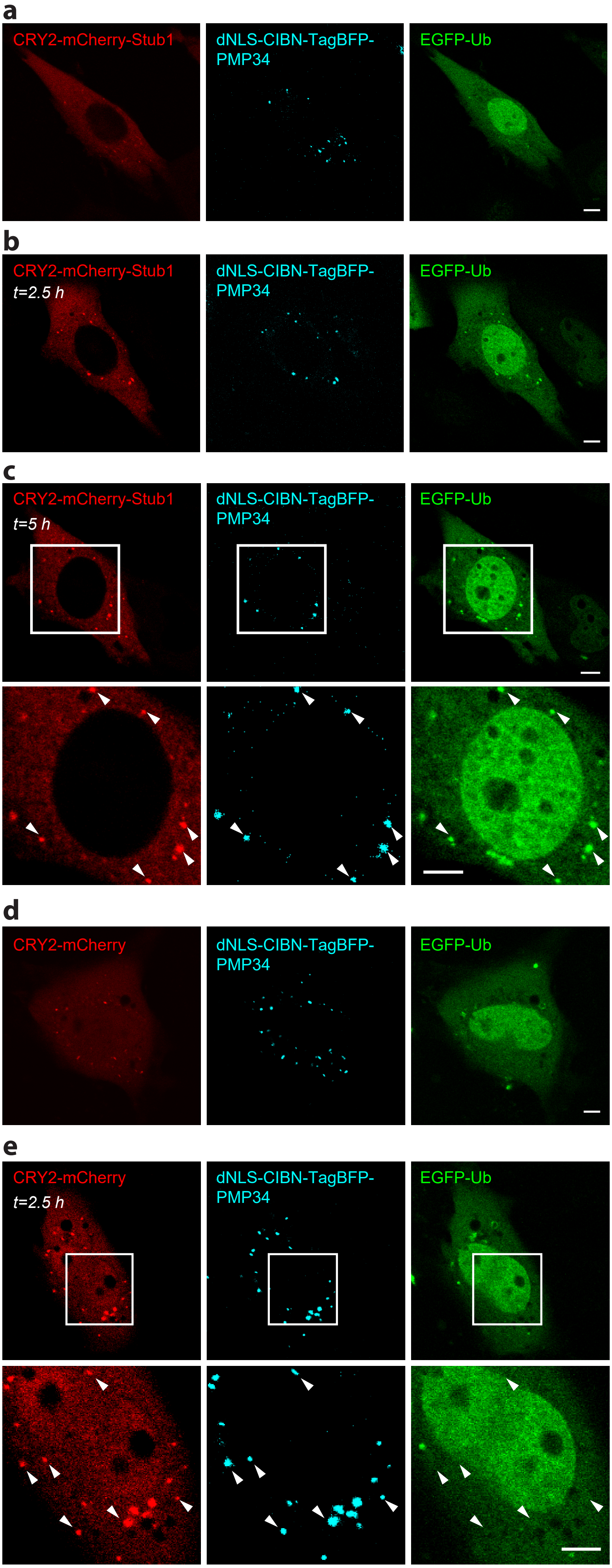

Supplementary Figure 4: Stub1-mediated peroxisomal ubiquitination.

A NIH3T3 cell expressing CRY2-mCherry-Stub1, dNLS-CIBN-TagBFP-PMP34 and EGFP-Ub was whole cell illuminated with 488 nm (19 µW, 2 min) (**a**), leading to CRY2-mCherry-Stub1 translocation onto peroxisomes. This led to the observed EGFP-Ub accumulation on peroxisomes 2.5 (**b**) and 5 hours (**c**) after illumination. (**c**, bottom panels) Magnified view of the white square region in the top panels showing full colocalization between Ub (green), Stub1 (red) and dNLS-CIBN-TagBFP-PMP34 (cyan, white arrowheads; n=6 cells) **d-e** Targeting CRY2-mCherry alone onto peroxisomes did not trigger peroxisomal ubiquitination. **d** A NIH3T3 cell expressing CRY2-mCherry, EGFP-Ub and dNLS-CIBN-TagBFP-PMP34 was whole cell illuminated with 488 nm (19 µW, 2 min), leading to CRY2-mCherry translocation onto dNLS-CIBN-TagBFP-PMP34 labeled peroxisomes. **e** However, no EGFP-Ub accumulation was seen 2.5 hours after illumination (bottom panels: magnified view of the white square region in the top panels, n=4 cells). All scale bars: 5 µm.

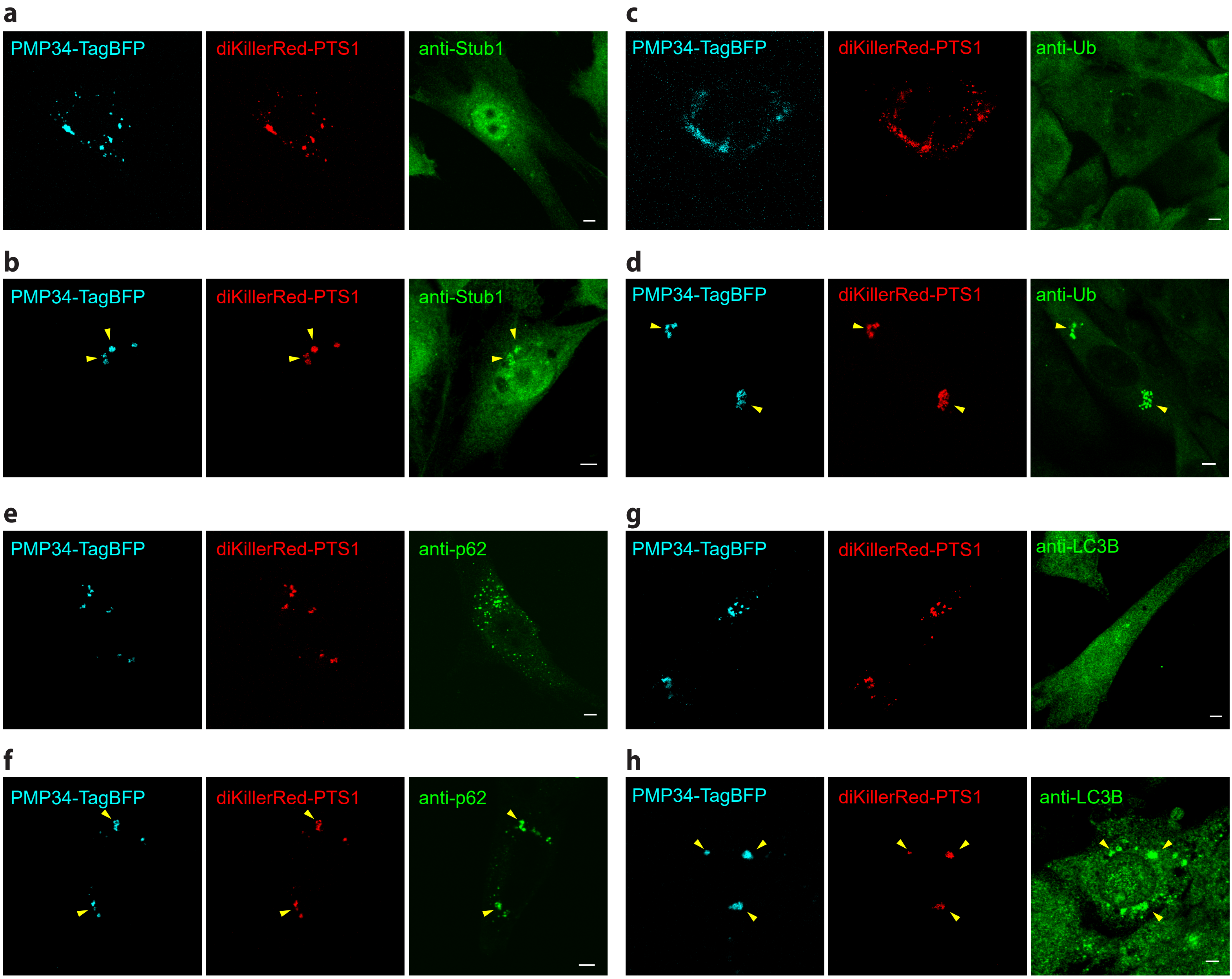

Supplementary Figure 5: Stub1-mediated pexophagy probed through immunofluorescence.
NIH3T3 cells expressing diKillerRed-PTS1 and PMP34-TagBFP before (**a**, **c**, **e**, **g**) and after (**b**, **d**, **f**, **g**) 565 nm illumination (LED; THORLABS, M565L2 500 mA, 9 hours). Immunofluorescence revealed that endogenous Stub1 (**b**), Ubiquitin (**d**), p62 (**f**), and LC3B (**h**) all accumulated on peroxisomes in 565 nm illuminated cells (yellow arrowheads). All scale bars: 5 µm.

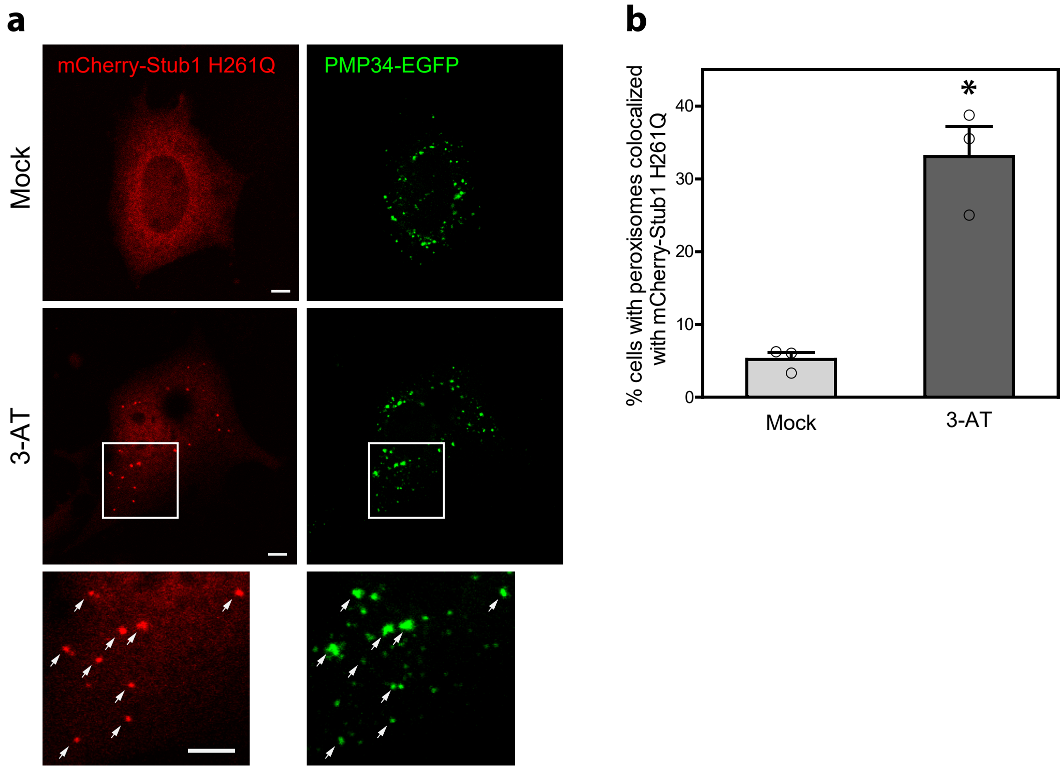

Supplementary Figure 6: Catalase inhibition resulted in Stub1 accumulation on peroxisomes.

a The mCherry-Stub1 H261Q accumulated on peroxisomes in catalase inhibitor 3-AT treated NIH3T3 cells (middle panels, 150 mM for 14 hours, n=20 cells; bottom panels, magnified view of the white square region in middle panels). On the other hand, mCherry-Stub1 H261Q did not accumulated on peroxisomes in non-treated cells (top panels). All scale bars: 5 µm. **b** The percentage of cells with more than 3 peroxisomes colocalized with mCherry-Stub1-H261Q (Mock: n=32, 30 and 33 cells; 3-AT: n=31, 31 and 28 cells. *P=0.0013 (one-tailed t-test)). All error bars represent the mean values + SEM. Source data are provided as a Source Data file.

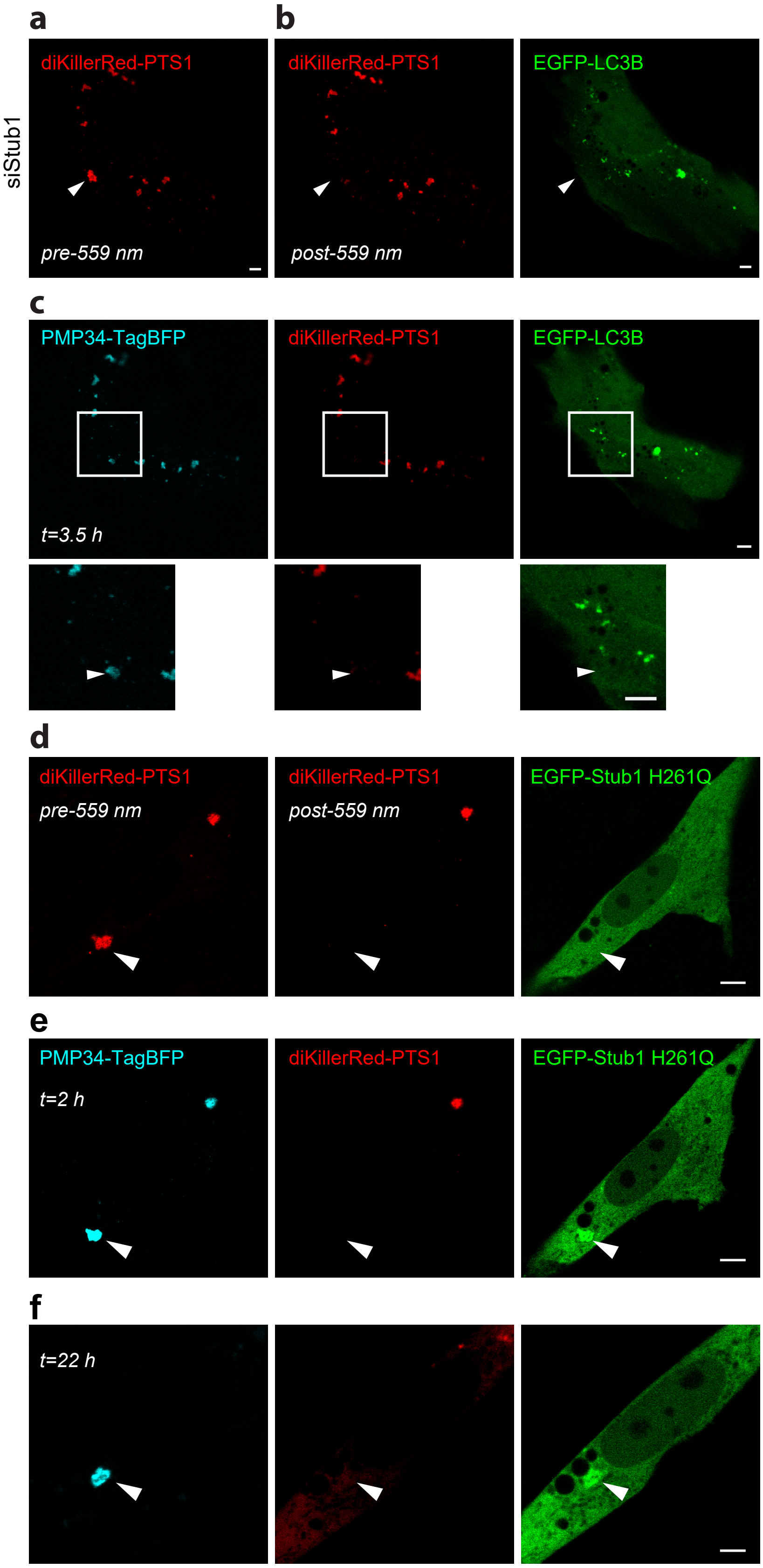

Supplementary Figure 7: Stub1 depletion or Stub1 H261Q overexpression blocks pexophagy.

a Specific peroxisomes (indicated by the white arrow) in a NIH3T3 cell transfected with diKillerRed-PTS1, PMP34-TagBFP, EGFP-LC3B, and siRNA against Stub1 (siStub1) were 559 nm illuminated (**b**, white arrowhead), leading to the immediate loss in diKillerRed-PTS1 fluorescence. **c** EGFP-LC3B did not accumulate onto ROS-stressed peroxisomes 3.5 hours after illumination. Bottom panels: magnified view of the white square in (**c**). **d-f** Overexpressing Stub1 ligase dead mutant H261Q blocks cellular turnover of ROS-stressed peroxisomes. **d** Specific peroxisomes in a NIH3T3 cell expressing diKillerRed-PTS1, PMP34-TagBFP and EGFP-Stub1 H261Q was 559 nm illuminated (left panel, white arrowhead), leading to the immediate loss in their diKillerRed-PTS1 fluorescence (middle and right panels). **e** ROS-stressed peroxisomes (white arrowhead, lacks diKillerRed-PTS1 fluorescence) remained detectable 2 hours and 22 hours (**f**) after 559 nm illumination (n=2 cells). All scale bars: 5 µm.

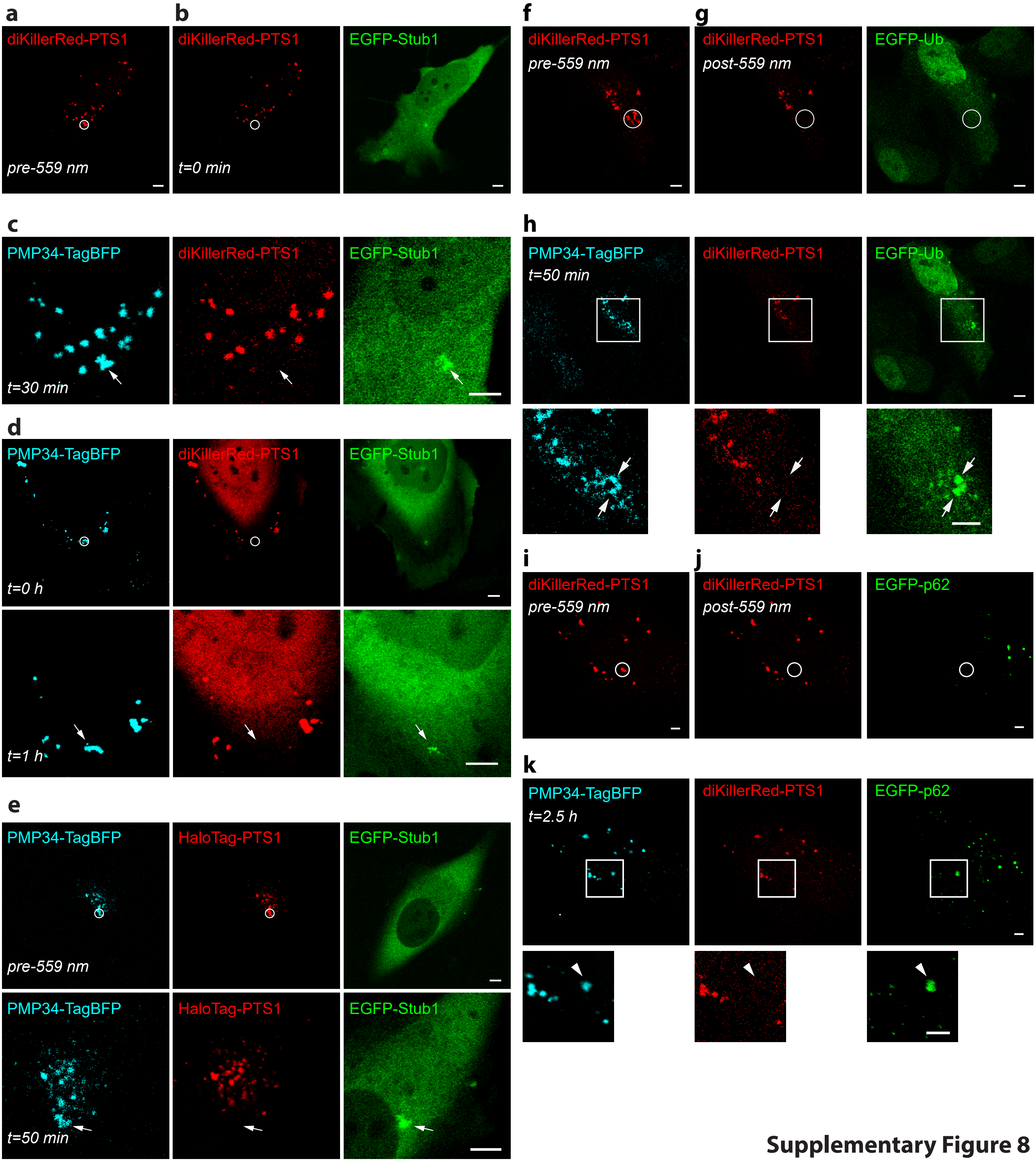

Supplementary Figure 8

Supplementary Figure 8: Stub1-mediated pexophagy in human cell lines.

A HeLa cell expressing diKillerRed-PTS1, PMP34-TagBFP and EGFP-Stub1 was 559 nm illuminated at the white circular region (**a**), leading to the immediate loss of diKillerRed-PTS1 fluorescence (**b**). Eventually EGFP-Stub1 accumulated on ROS-stressed peroxisomes (**c**, white arrows, n=11 cells). **d** As in (**a-b**), a HeLa cell right after 559 nm illumination (top panels). Eventually EGFP-Stub1 accumulated on ROS-stressed peroxisomes (bottom panels, white arrows). **e** A U2OS cell expressing HaloTag-PTS1, PMP34-TagBFP and EGFP-Stub1 was stained with the HaloTag TMR ligand (100 nM, 40 min), and 559 nm illuminated at the white circular region (top panels). Eventually EGFP-Stub1 accumulated on ROS-stressed peroxisomes (bottom panels, white arrows, n=10 cells). A HeLa cell expressing diKillerRed-PTS1, PMP34-TagBFP and EGFP-Ub was 559 nm illuminated at the white circular region (**f**), leading to the immediate loss of diKillerRed-PTS1 fluorescence (**g**). Eventually EGFP-Ub accumulated on ROS-stressed peroxisomes (**h**, white arrows, n=8 cells). A HeLa cell expressing diKillerRed-PTS1, PMP34-TagBFP and EGFP-p62 was 559 nm illuminated at the white circular region (**i**), leading to the immediate loss of diKillerRed-PTS1 fluorescence (**j**). Eventually EGFP-p62 accumulated on ROS-stressed peroxisomes (**k**, white arrows, n=7 cells). Bottom panels: magnified view of the white square region in the middle panels. All scale bars: 5 µm.

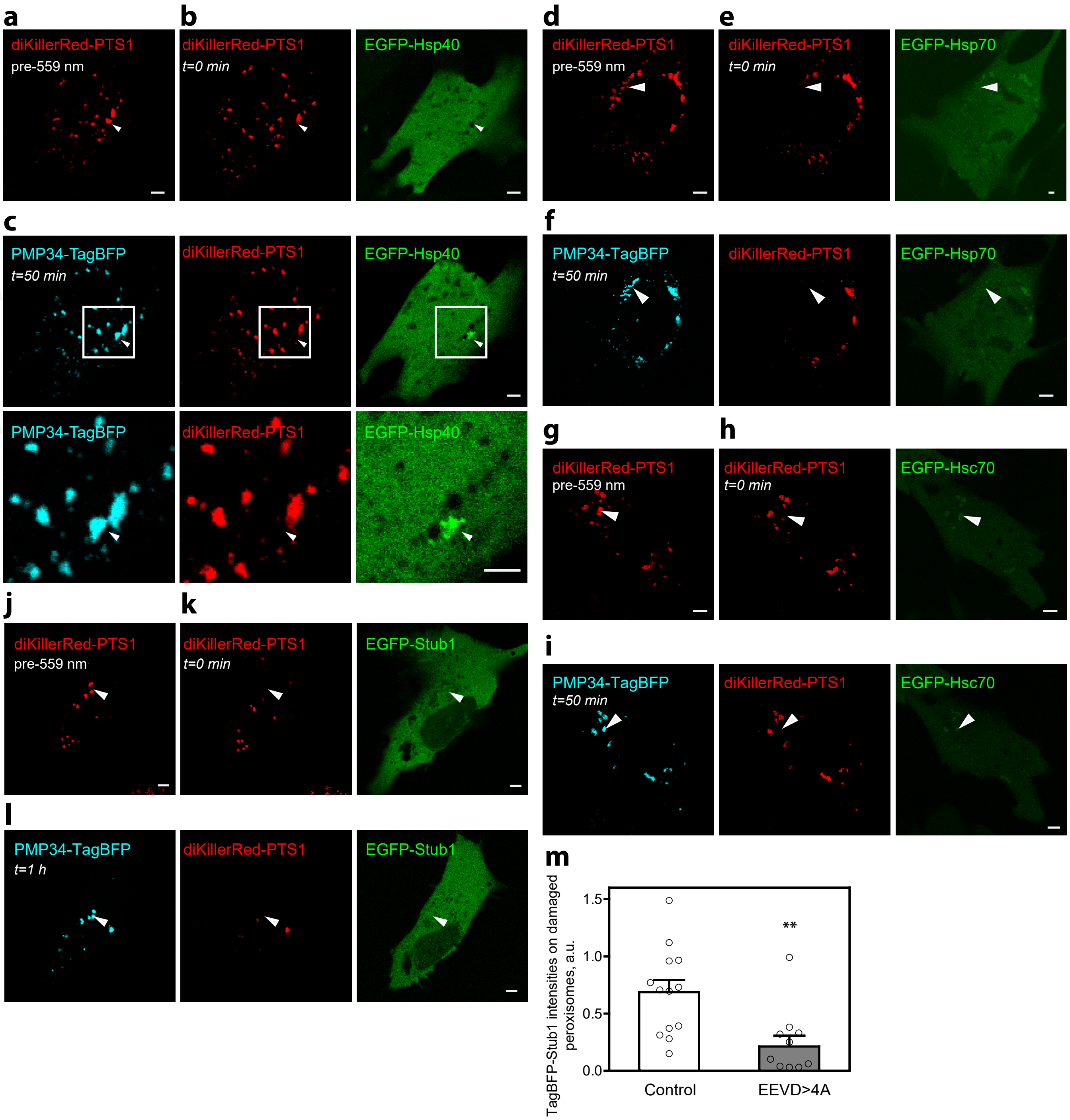

Supplementary Figure 9: Heat-shock proteins in pexophagy.

a-c Hsp40 accumulates on ROS-stressed peroxisomes. **a** The peroxisomes indicated by the white arrowhead in a NIH3T3 cell expressing EGFP-Hsp40, diKillerRed-PTS1 and PMP34-TagBFP were 559 nm illuminated, leading to the immediate loss of diKillerRed-PTS1 fluorescence (**b**). Later (50 minutes after illumination shown) EGFP-Hsp40 accumulated on ROS-stressed peroxisomes (**c**, top panels, white arrowheads, n=7 cells). Bottom panels: magnified view of white square region in the top panels (white arrowheads indicate ROS-stressed peroxisomes). **d-l** PES-Cl inhibits Hsp70, Hsc70 and Stub1 accumulations on ROS-stressed peroxisomes. The peroxisomes indicated by the white arrowhead in a PES-Cl treated NIH3T3 cell expressing diKillerRed-PTS1, PMP34-TagBFP and EGFP-Hsp70 was 559 nm illuminated (**d**), leading to the immediate loss of diKillerRed-PTS1 fluorescence (**e**). PES-Cl blocked EGFP-Hsp70 accumulation on ROS-stressed peroxisomes (**f**, white arrowheads, n=10 cells). The peroxisomes indicated by the white arrowhead in a PES-Cl treated NIH3T3 cell expressing diKillerRed-PTS1, PMP34-TagBFP and EGFP-Hsc70 was 559 nm illuminated (**g**), leading to the immediate loss of diKillerRed-PTS1 fluorescence (**h**). PES-Cl blocked EGFP-Hsc70 accumulation on ROS-stressed peroxisomes (**i**, white arrowheads, n=12 cells). The peroxisomes indicated by the white arrowhead in a PES-Cl treated NIH3T3 cell expressing diKillerRed-PTS1, PMP34-TagBFP and EGFP-Stub1 was 559 nm illuminated (**J**), leading to the immediate loss of diKillerRed-PTS1 fluorescence (**k**). PES-Cl blocked EGFP-Stub1 accumulation on ROS-stressed peroxisomes (**l**, white arrowheads, n=6 cells). **m** Quantifying TagBFP-Stub1 accumulation on ROS-stressed peroxisomes (in NIH3T3 cells expressing diKillerRed-PTS1, PMP34-EGFP and TagBFP-Stub1). Overexpressing EGFP-Hsp70 EEVD>4A reduced TagBFP-Stub1 accumulation (50 min after 559 nm illumination, n=13 and 11 cells) **P=0.0016 (one-tailed t-test). All error bars represent the mean values + SEM. All scale bars: 5 µm. Source data are provided as a Source Data file.

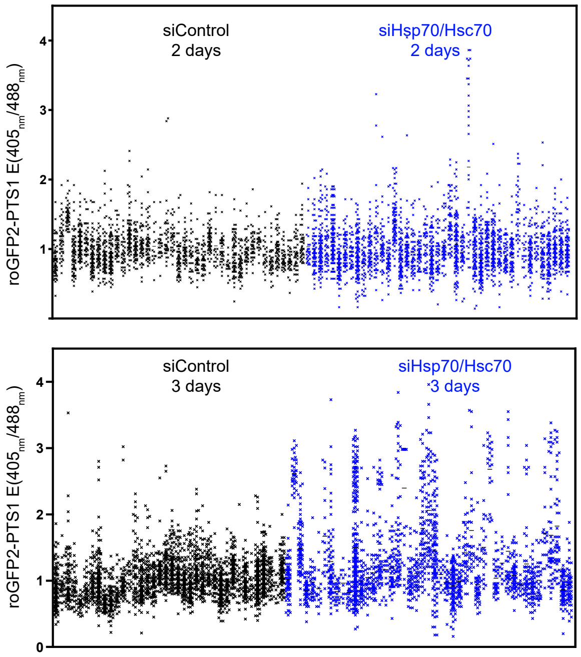

Supplementary Figure 10: Hsc70/Hsp70 depletion led to cellular accumulation of oxidatively-stressed peroxisomes. Each dot within the plots represents roGFP2-PTS1 emission ratio (between 405 nm and 488 nm excitation) of one peroxisome, and a column denotes all peroxisomes within a single cell. The ratios for Hsc70/Hsp70 depleted cells vs. that of control cells are shown (top: 48 hrs after depletion; bottom: 72 hrs after depletion). Source data are provided as a Source Data file.

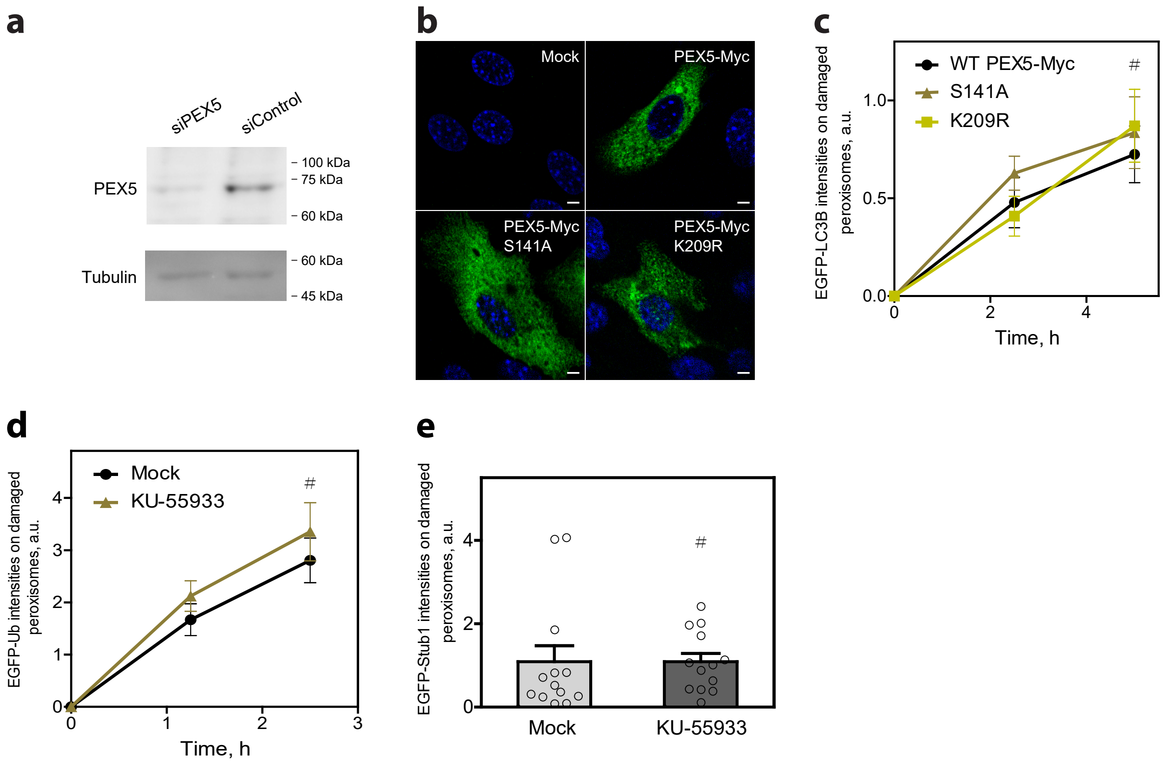

Supplementary Figure 11: The effects of ATM inhibition on this pexophagy.

a Efficiency of the PEX5 siRNA utilized in this paper (3 days after transfection). **b** The expressions of PEX5-Myc (wild-type or the indicated mutants) were immunostained with the anti-Myc antibody 3 days after transfection. All scale bars: 5 µm. **c** The accumulation of EGFP-LC3B on the 559 nm light illuminated peroxisomes in NIH3T3 cells transfected with EGFP-LC3B, PMP34-TagBFP, diKillerRed-PTS1, PEX5 siRNA, and wild-type PEX5-Myc or the indicated PEX5 mutants 3 days after transfection. (n=19, 17 and 11 cells. $^{\#}$P=0.3169 (WT vs. S141A) P=0.2731 (WT vs. K209R) (one-tailed t-test)). **d** The NIH3T3 cells were transfected with EGFP-Ub, PMP34-TagBFP and diKillerRed-PTS1 and pretreated with the ATM inhibitor KU-55933 (500 nM, 2hrs) before 559 nm illumination. The accumulations of EGFP-Ub on 559 nm light illuminated peroxisomes were quantified (n=16 and 17 cells respectively, $^{\#}$P=0.2225 (one-tailed t-test)). All error bars in (**c**-**d**) represent the mean values ± SEM. **e** The effect of the ATM inhibitor KU-55933 (500 nM, 2 hrs) on EGFP-Stub1 accumulation were determined (in non-treated or KU-55933 pretreated NIH3T3 cells expressing diKillerRed-PTS1, PMP34-TagBFP and EGFP-Stub1) by quantifying the translocated EGFP-Stub1on ROS-stressed peroxisomes (50 min after 559 nm illumination, n=13 and 13 cells $^{\#}$P=0.4993 (one-tailed t-test)). All error bars represent the mean values + SEM. Source data are provided as a Source Data file.

Supplementary Tables

a

Target Gene	Target Sequence	Genomic Location
Stub1	AGCGATCAGCTCCGCCCGGTCGG	Ch.17: +25832913 – +25832935
Stub1	GCGAGCGAGCCATCGAGTCCAGG	Ch.17: +25832595 – +25832617

b

sgRNA Template	Template Sequence
mStub1 sgRNA 1	TAA TAC GAC TCA CTA T AGCGATCAGCTCCGCCCGGT GT TTT AGA GCT ATG CTG GAA ACA GCA TAG CAA GTT AAA ATA AGG CTA GTC CGT TAT CAA CTT GAA AAA GTG GCA CCG AGT CGG TGC
mStub1 sgRNA 2	TAA TAC GAC TCA CTA TA GCGAGCGAGCCATCGAGTCC GT TTT AGA GCT ATG CTG GAA ACA GCA TAG CAA GTT AAA ATA AGG CTA GTC CGT TAT CAA CTT GAA AAA GTG GCA CCG AGT CGG TGC

Supplementary Table 1: Designs for generating Stub1 knockout cells.

a Target sequences chosen for generating Stub1 knockout NIH3T3 cells. **b** The sgRNAs were used in generating Stub1 knockout NIH3T3 cells (each including a T7 promoter, a 20 nt target sequence (without PAM), and a published sgRNA scaffold in blue).

Constructs	Primers	Sequences (5'→3')
PMP34-TagBFP		
	NheI-PMP34-F	GCGAGCGCTAGCATGGCTTCCGTGCTGTCCTACG
	PMP34-NheI-R	GCACTCGCTAGCCCGGAACCGCCTCCGTGTTGGTGTGCACGCTTCAGC
EGFP-Stub1		
	XhoI-Stub1-F	CGCGCGCTCGAGGCATGAAGGGCAAGGAGGAAAAGGA
	Stub1-XmaI-R	CGCGCGCCCGGGTTAATAGTCCTCTACCCAGCCGTTCTCAG
diKillerRed-PTS1		
	KpnI-Vec-F	GCGCGCGGTACCGCGGGCCCGG
	KillerRed Vec-R	CTACCGCTACCTGTGCTACCTGTGCCATGATCCTCGTCGCTACCGATGGC
	KillerRed-F	CAGCGGTACAGCGTCTAGCGAGGATAATAACATGGCTATGGGTTCAGAGGGCGGCC
	KillerRed-R	GCGCGCGGTACCTTATCAATCCTCGTCGCTACCGATGGC
	VKSKL-F	GTCAAGTCGAAGCTCTAAAGATAACTGATCATAATCAGCCATACCACAT
	VKSKL-R	AGATCCGGTGGATCCCGGG
EGFP-Hsp70		
	Hsp70-EcoRI-F	GCGCGCGAATTCTGGAGGAGGATCTATGGCCAAAGCCGCGGC
	Hsp70-BamHI-R	GCGCGCGGATCCTTAATCTACCTCCTCAATGGTGGGGCC
roGFP2-PTS1		
	PTS1 Vector-F	GTCAAGTCGAAGCTCTAAAGATAACTGATCATAATCAGCCATACCACAT
	Vector-AgeI-R	GCGCGCACCGGTAGCGCTAGCGGATCT
	AgeI-roGFP2-F	GCGCGCACCGGTATGTTTTTCAACAGACTAAGCGCTGG
	roGFP2-R	CAATTCGTCGTGCTTGTACAATTCG
PMP34-PAGFP		
	NheI-PMP34-F	GCGAGCGCTAGCATGGCTTCCGTGCTGTCCTACG
	PMP34-AgeI-R	CGCGCGACCGGTCCGTGTTGGTGTGCACGCTTCAGC
CRY2-mCherry-Stub1		
	XhoI-Stub1-F	CGCGCGCTCGAGGCATGAAGGGCAAGGAGGAAAAGGA
	Stub1-XmaI-R	CGCGCGCCCGGGTTAATAGTCCTCTACCCAGCCGTTCTCAG
dNLS-CIBN-TagBFP-PMP34		
	NheI-CIBN-F	GCGCGCGGCTAGCATGAATGGAGCTATAGGAGGTGACCTTT
	CIBN-NheI-R	GCGCCCGCTAGCCCGGACCCACCACCTCCAGAGC
	delete NLS-F	AGACTAAGGATTGTAATGAGGCGGCGAAGAAGATGACGATGAACAGAGATGACC
	delete NLS-R	CTGTATCAAACTTCGCTGCCTTGAAATTTCCAGTCCCAAGCGT
	SalI-KpnI-PMP34-F	GCGCGCGTCGACGGTACCATGGCTTCCGTGCTGTCCTACG
	PMP34-BamHI-R	GCGCGCGGATCCTCAGTGTTGGTGTGCACGCTTCAGC
TagBFP-hStub1		
	EcoRI-hStub1-F	GCGCGCGAATTCAGGTGGTTCAGGTGGTGGATCTGGTATGAAGGGCAAGGAGGAGAAGGA
	hStub1-BamHI-R	GCGCGCGGATCCTTAGTAGTCCTCCACCCAGCCATTCTC
EGFP-p62		
	EcoRI-p62-F	GCGCGCGAATTCTATGGCGTCGCTCACCGTGA
	p62-KpnI-R	GCGCGCGGTACCTCACAACGGCGGGGGATGCT
EGFP-Hsp40		
	XhoI-DNAJA1-F	GCGCGCGCGCTCGAGCTATGGTGAAAGAAACAACTTACTACGATGTTT
	DNAJA1-BamHI-R	GCGCGCGCGGATCCCTAAGAGGTCTGACACTGAACACCACCTC
HaloTag-PTS1		
	Halo-PTS1-F	CGAGCTGTTGAACTAGATCCTGAGTTTG
	Halo-PTS1-R	CTACTATAGTTTAGACTTAACAGATCTGAGTCCGGAGCCGGA
PMP34-EGFP		
	NheI-PMP34-F	GCGAGCGCTAGCATGGCTTCCGTGCTGTCCTACG
	PMP34-AgeI-R	CGCGCGACCGGTCCGTGTTGGTGTGCACGCTTCAGC
PMP34-EGFP-TagBFP2		
	34GB-F	GGTGGAAGCGCCACCATGAGCGAGCTG
	34GB-R	ACCGGTGGATCCCGGGCC
mCherry-Stub1 H261Q		
	delCRY2-F	ATGGTGAGCAAGGGCGAGGA
	delCRY2-R	CATGCTAGCGGATCTGACGGTTC
mutagenesis		
	H261Q-F	CAGCTGCAGCGTGTGGGCCACT
	H261Q-R	CTCCTCAATGTCCTTGCGGTCAT
	K31A-F	GCAGAGCAGGGAAACCGGCTCTTC
	K31A-R	GAGCTCTTGCGCACTCGGG
	EEVD>4A-F	GGATCCACCGGATCTAGATAACTGATC
	EEVD>4A-R	CTAGGCAGCCGCAGCAATGGTGGGGCCTGACCCA
	N65S-F	TCTCGGGCCTTGTGCTACCTGAAG
	N65S-R	GGTGTAATACACGGCCACCAGC
	A79D-F	CCTGGCCGACTGCCGG
	A79D-R	TCCTGCTCGTGCTGCTGCATC
	L123V-F	TGGCCAAGGAGCAGCGGC
	L123V-R	CGCTGTAAGCTCGCTGCAGATTGG
	K144X-F	AGAAGCGCTGGAACAGCATTGAG
	K144X-R	ACTTCGCGATTCGAAGAGCGC
	M240T-F	GCGGGAGCCGTGCATCA
	M240T-R	GTCAGCTCAAAGCTGATCTTGCCA
	T246M-F	GCCCAGTGGCATCACCTACGA
	T246M-R	ATGATGCACGGCTCCCGCA
PEX5-Myc		
	PEX5-F	GCGAGCGCGCTAGCATGGCAATGCGGGAGCTGG
	PEX5-R	GCACGCGCGCTAGCTCACTGGGGCAGGCCAAACATAG
	Myc-F	CTCATTTCTGAAGAGGACTTGAATGAGCTAGCGGTCGCCACCAT
	Myc-R	CTTTTGCTCCATAGAACCAGAACCCTGGGGCAGGCCAAACATAGTTA
	TAGa-F	AATGAGCTAGCGGTCGCCACC
	TAGa-R	TCTACAAGTCCTCTTCAGAAATGAGCTTTTG
	S141A-F	GCCCAAGAATTCATCTCTGAAGTTACAGACC
	S141A-R	CCAGTCAGTCTCATTATAATCCTGAGTTACATC
	K209R-F	GATTGGCTAATTCTGAGTTCCTGAAATTCG
	K209R-R	TGGGGTCATCCACTTTGGCCACAA

Supplementary Table 2: Primers used for plasmid construction
