# Supplementary material for: Hsc70/Stub1 promotes the removal of individual oxidatively stressed peroxisomes
Source: Nat Commun. 2020 Oct 19;11:5267. doi: 10.1038/s41467-020-18942-3 (PMC7573593; doi:10.1038/s41467-020-18942-3)
Supplement: Supplementary file 2 — Reporting Summary [file 41467_2020_18942_MOESM2_ESM.pdf]

nature research

Corresponding author(s): Wei Yuan Yang

Last updated by author(s): Aug 27, 2020

Reporting Summary

Nature Research wishes to improve the reproducibility of the work that we publish. This form provides structure for consistency and transparency in reporting. For further information on Nature Research policies, see Authors & Referees and the Editorial Policy Checklist.

Statistics

For all statistical analyses, confirm that the following items are present in the figure legend, table legend, main text, or Methods section.

n/a	Confirmed	
☐	☒	The exact sample size (*n*) for each experimental group/condition, given as a discrete number and unit of measurement
☐	☒	A statement on whether measurements were taken from distinct samples or whether the same sample was measured repeatedly
☐	☒	The statistical test(s) used AND whether they are one- or two-sided<br>*Only common tests should be described solely by name; describe more complex techniques in the Methods section.*
☒	☐	A description of all covariates tested
☒	☐	A description of any assumptions or corrections, such as tests of normality and adjustment for multiple comparisons
☐	☒	A full description of the statistical parameters including central tendency (e.g. means) or other basic estimates (e.g. regression coefficient) AND variation (e.g. standard deviation) or associated estimates of uncertainty (e.g. confidence intervals)
☐	☒	For null hypothesis testing, the test statistic (e.g. *F*, *t*, *r*) with confidence intervals, effect sizes, degrees of freedom and *P* value noted<br>*Give P values as exact values whenever suitable.*
☒	☐	For Bayesian analysis, information on the choice of priors and Markov chain Monte Carlo settings
☒	☐	For hierarchical and complex designs, identification of the appropriate level for tests and full reporting of outcomes
☒	☐	Estimates of effect sizes (e.g. Cohen's *d*, Pearson's *r*), indicating how they were calculated

Our web collection on statistics for biologists contains articles on many of the points above.

Software and code

Policy information about availability of computer code

Data collection	Olympus FluoView FV1000
Data analysis	ImageJ 1.48v, Excel 2013, and GraphPad Prism 5

For manuscripts utilizing custom algorithms or software that are central to the research but not yet described in published literature, software must be made available to editors/reviewers. We strongly encourage code deposition in a community repository (e.g. GitHub). See the Nature Research guidelines for submitting code & software for further information.

Data

Policy information about availability of data

All manuscripts must include a data availability statement. This statement should provide the following information, where applicable:

- Accession codes, unique identifiers, or web links for publicly available datasets
- A list of figures that have associated raw data
- A description of any restrictions on data availability

The data that support the findings of this study are available from the corresponding author upon reasonable request.

Field-specific reporting

Please select the one below that is the best fit for your research. If you are not sure, read the appropriate sections before making your selection.

☒ Life sciences ☐ Behavioural & social sciences ☐ Ecological, evolutionary & environmental sciences

For a reference copy of the document with all sections, see nature.com/documents/nr-reporting-summary-flat.pdf

Life sciences study design

All studies must disclose on these points even when the disclosure is negative.

Sample size	Sample sizes were chosen based on our experiences with the assays performed. Sample sizes are clearly stated in the figure legends.
Data exclusions	No data were excluded.
Replication	The number of replications for each experiment are clearly stated in the figure legends. Most data in the paper are from long-term single cell imaging/manipulation assays, and the number of cells imaged/manipulated represent the number of times an experiment was performed.
Randomization	All experiments described in this work are based on well established cell lines. Samples and treatments are all of defined compositions. Randomization was therefore not performed.
Blinding	The experiments were not blinded as all experiments were carried out single-handedly by the first author. Use of defined procedures for software-based image analysis (in ImageJ) minimized the occurrence of bias.

Behavioural & social sciences study design

All studies must disclose on these points even when the disclosure is negative.

Study description	*Briefly describe the study type including whether data are quantitative, qualitative, or mixed-methods (e.g. qualitative cross-sectional, quantitative experimental, mixed-methods case study).*
Research sample	*State the research sample (e.g. Harvard university undergraduates, villagers in rural India) and provide relevant demographic information (e.g. age, sex) and indicate whether the sample is representative. Provide a rationale for the study sample chosen. For studies involving existing datasets, please describe the dataset and source.*
Sampling strategy	*Describe the sampling procedure (e.g. random, snowball, stratified, convenience). Describe the statistical methods that were used to predetermine sample size OR if no sample-size calculation was performed, describe how sample sizes were chosen and provide a rationale for why these sample sizes are sufficient. For qualitative data, please indicate whether data saturation was considered, and what criteria were used to decide that no further sampling was needed.*
Data collection	*Provide details about the data collection procedure, including the instruments or devices used to record the data (e.g. pen and paper, computer, eye tracker, video or audio equipment) whether anyone was present besides the participant(s) and the researcher, and whether the researcher was blind to experimental condition and/or the study hypothesis during data collection.*
Timing	*Indicate the start and stop dates of data collection. If there is a gap between collection periods, state the dates for each sample cohort.*
Data exclusions	*If no data were excluded from the analyses, state so OR if data were excluded, provide the exact number of exclusions and the rationale behind them, indicating whether exclusion criteria were pre-established.*
Non-participation	*State how many participants dropped out/declined participation and the reason(s) given OR provide response rate OR state that no participants dropped out/declined participation.*
Randomization	*If participants were not allocated into experimental groups, state so OR describe how participants were allocated to groups, and if allocation was not random, describe how covariates were controlled.*

Ecological, evolutionary & environmental sciences study design

All studies must disclose on these points even when the disclosure is negative.

Study description	*Briefly describe the study. For quantitative data include treatment factors and interactions, design structure (e.g. factorial, nested, hierarchical), nature and number of experimental units and replicates.*
Research sample	*Describe the research sample (e.g. a group of tagged Passer domesticus, all Stenocereus thurberi within Organ Pipe Cactus National Monument), and provide a rationale for the sample choice. When relevant, describe the organism taxa, source, sex, age range and any manipulations. State what population the sample is meant to represent when applicable. For studies involving existing datasets, describe the data and its source.*
Sampling strategy	*Note the sampling procedure. Describe the statistical methods that were used to predetermine sample size OR if no sample-size calculation was performed, describe how sample sizes were chosen and provide a rationale for why these sample sizes are sufficient.*
Data collection	*Describe the data collection procedure, including who recorded the data and how.*
Timing and spatial scale	*Indicate the start and stop dates of data collection, noting the frequency and periodicity of sampling and providing a rationale for*

Timing and spatial scale | *these choices. If there is a gap between collection periods, state the dates for each sample cohort. Specify the spatial scale from which the data are taken*

Data exclusions | *If no data were excluded from the analyses, state so OR if data were excluded, describe the exclusions and the rationale behind them, indicating whether exclusion criteria were pre-established.*

Reproducibility | *Describe the measures taken to verify the reproducibility of experimental findings. For each experiment, note whether any attempts to repeat the experiment failed OR state that all attempts to repeat the experiment were successful.*

Randomization | *Describe how samples/organisms/participants were allocated into groups. If allocation was not random, describe how covariates were controlled. If this is not relevant to your study, explain why.*

Blinding | *Describe the extent of blinding used during data acquisition and analysis. If blinding was not possible, describe why OR explain why blinding was not relevant to your study.*

Did the study involve field work? ☐ Yes ☐ No

Field work, collection and transport

Field conditions | *Describe the study conditions for field work, providing relevant parameters (e.g. temperature, rainfall).*

Location | *State the location of the sampling or experiment, providing relevant parameters (e.g. latitude and longitude, elevation, water depth).*

Access and import/export | *Describe the efforts you have made to access habitats and to collect and import/export your samples in a responsible manner and in compliance with local, national and international laws, noting any permits that were obtained (give the name of the issuing authority, the date of issue, and any identifying information).*

Disturbance | *Describe any disturbance caused by the study and how it was minimized.*

Reporting for specific materials, systems and methods

We require information from authors about some types of materials, experimental systems and methods used in many studies. Here, indicate whether each material, system or method listed is relevant to your study. If you are not sure if a list item applies to your research, read the appropriate section before selecting a response.

Materials & experimental systems

n/a	Involved in the study
☐	☒ Antibodies
☐	☒ Eukaryotic cell lines
☒	☐ Palaeontology
☒	☐ Animals and other organisms
☒	☐ Human research participants
☒	☐ Clinical data

Methods

n/a	Involved in the study
☒	☐ ChIP-seq
☒	☐ Flow cytometry
☒	☐ MRI-based neuroimaging

Antibodies

Antibodies used |
anti-Hsp70/HspA1A (R&D system, MAB1663 mouse monoclonal to Hsp70)
anti-Hsc70/HspA8 (Abcam, ab2788 mouse monoclonal to Hsc70)
anti-Stub1 (Abcam, ab134064 rabbit monoclonal to Stub1)
anti-Ubiquitin (Sigma-Aldrich, 04-263 mouse monoclonal (FK2) to mono- and poly ubiquitinylated proteins)
anti-p62 (Abcam, ab56416 mouse monoclonal to Sqstm1)
anti-LC3B (Novus Biologicals, NB100-2220 rabbit polyclonal to LC3B)
anti-ATG5 (GeneTex, GTX62601 rabbit monoclonal to ATG5)
anti-tubulin (Abcam, ab6160 rat monoclonal [YL1/2] to tubulin)
anti-PEX5 (Novus Biologicals, NBP1-87185 rabbit polyclonal to PEX5)
anti-Myc antibody (Cell Signaling, 2278 rabbit monoclonal to Myc-tag)
Alexa Fluor 488 goat anti rabbit IgG (H+L) (Thermo Fisher scientific, A-11034 polyconal to rabbit IgG)
Alexa Fluor 488 goat anti mouse IgG (H+L) (Thermo Fisher scientific, A-11029 polyconal to mouse IgG)

Validation |
Validation statements of all the antibodies used are provided on the manufacturers' websites.

anti-Hsp70/HspA1A (R&D system, MAB1663): Lysates of Jurkat human acute T cell leukemia cell line and NIH-3T3 mouse embryonic fibroblast cell line were used for western blot validation. A specific band was detected for HSP70/HSPA1A at approximately 70 kDa. Species reactivity: Human, Mouse, Rat

anti-Hsc70/HspA8 (Abcam, ab2788): validated Western blot using NIH-3T3 cell lysate on the manufacturers' websites. (https://www.abcam.com/hsc70-antibody-13d3-ab2788.html) Immunohistochemistry for human prostate carcinoma tissue and

immunofluorescence assay for HeLa cells were validated. Species reactivity: Human, Mouse, Rat

anti-Stub1 (Abcam, ab134064): It were validated for western blot of lysates from the HeLa cells, MCF7 cells and HEK293 cells. Immunofluorescence staining of SH-SY5Y cells and immunohistochemical analysis of paraffin-embedded Human skeletal muscle tissue were also validated. Species reactivity: Human, Mouse, Rat

anti-Ubiquitin (Sigma-Aldrich, 04-263 mouse monoclonal (FK2) to mono- and poly ubiquitinylated proteins)
It has been validated for use in ELISA, Immunofluorescence (IF), Immunoprecipitation (IP) and Western Blotting (WB) for the detection of Ubiquitinylated proteins. Species Reactivity: All

anti-p62 (Abcam, ab56416): Western blots for Hap1 and HeLa cell lysates were validated. Immunofluorescence analysis of HeLa cells was validated. Staining SQSTM1 in human lymph node was validated for immunohistochemistry. Species reactivity: Human, Mouse (predicted)

anti-LC3B (Novus Biologicals, NB100-2220 rabbit polyclonal to LC3B)
Applications for WB, ELISA, Flow,ICC/IF, IHC, IP were valited. Species Reactivity: Hu, Mu, Rt, Po, Av, Ba, Bv, Ca, Ch, Gp, Ha, In, Pm, Pm, Rb, SyHa

anti-ATG5 (GeneTex, GTX62601): It was validated for Western blot detection for NT2D1, PC-3 and HeLa cell lysates. Immunofluorescent analysis for HeLa cells was also validated. Species reactivity: Human, Mouse

anti-tubulin (Abcam, ab6160): It was validated for Western blot detection for HeLa, NIH3T3, PC-12 and BALB/3T3 whole cell lysates. Immunofluorescent analysis for HeLa cells was also validated. Species reactivity: Human, Mouse, Pig

anti-PEX5 (Novus Biologicals, NBP1-87185): Western blot analysis in mouse cell line NIH3T3 and rat cell line NBT-II was validated. Immunofluorescence staining of human cell line A-431 was validated. Species reactivity: Human, Mouse, Rat

anti-Myc antibody (Cell Signaling, 2278): Western blot analysis of extracts from untransfected control cells and transfected cells overexpressing Myc-Bcl-2 was validated. Immunofluorescent analysis of 293 cells stably expressing Myc-tagged ADORA2A versus wild-type 293 cells was performed for validation. Species reactivity: All

Alexa Fluor 488 goat anti rabbit IgG (H+L) (Thermo Fisher scientific, A-11034): It was used for the immunocytochemistry analysis of HeLa cells stained with Tau (pT231) Recombinant Rabbit Monoclonal Antibody. It was also validated by detecting HeLa cells stained with alpha Tubulin Rabbit Polyclonal Antibody (Product # PA5-16891). Species reactivity: rabbit IgG

Alexa Fluor 488 goat anti mouse IgG (H+L) (Thermo Fisher scientific, A-11029): It was validated by performing immunofluorescence assay for HeLa cells stained with alpha Tubulin (236-10501) Mouse Monoclonal Antibody (Product # A11126). Species reactivity: mouse IgG

Eukaryotic cell lines

Policy information about cell lines

Cell line source(s)	HeLa and NIH3T3 cell lines were purchased from the American Type Culture Collection (ATCC). U2OS cell line was purchased from the Bioresource Collection and Research Center in Taiwan (BCRC, Hsinchu, Taiwan).
Authentication	Cell lines were not authenticated at the molecular level.
Mycoplasma contamination	Used cell lines were tested negative for mycoplasma contamination.
Commonly misidentified lines (See ICLAC register)	No cell lines used are listed in the ICLAC database.

Animals and other organisms

Policy information about studies involving animals; ARRIVE guidelines recommended for reporting animal research

Laboratory animals	*For laboratory animals, report species, strain, sex and age OR state that the study did not involve laboratory animals.*
Wild animals	*Provide details on animals observed in or captured in the field; report species, sex and age where possible. Describe how animals were caught and transported and what happened to captive animals after the study (if killed, explain why and describe method; if released, say where and when) OR state that the study did not involve wild animals.*
Field-collected samples	*For laboratory work with field-collected samples, describe all relevant parameters such as housing, maintenance, temperature, photoperiod and end-of-experiment protocol OR state that the study did not involve samples collected from the field.*
Ethics oversight	*Identify the organization(s) that approved or provided guidance on the study protocol, OR state that no ethical approval or guidance was required and explain why not.*

Note that full information on the approval of the study protocol must also be provided in the manuscript.
